# Supplementary material for: Polymorphic amyloid nanostructures of hormone peptides involved in glucose homeostasis display reversible amyloid formation
Source: Nat Commun. 2023 Aug 1;14:4621. doi: 10.1038/s41467-023-40294-x (PMC10394066; doi:10.1038/s41467-023-40294-x)
Supplement: Supplementary file 1 — Supplementary Information [file 41467_2023_40294_MOESM1_ESM.pdf]

Polymorphic Amyloid Nanostructures of Hormone Peptides Involved in Glucose Homeostasis Display Reversible Amyloid Formation

Supplementary Figures, Tables and Discussion

Dániel Horváth[1], Zsolt Dürvanger[1,2], Dóra K. Menyhárd[1,2], Máté Sulyok-Eiler[2,3], Fruzsina Bencs[2,3], Gergő Gyulai[4], Péter Horváth[5], Nóra Taricska[1] and András Perczel[1,2,*]

[1] ELKH-ELTE Protein Modeling Research Group ELTE Eötvös Loránd University, Pázmány Péter sétány 1/A, H-1117 Budapest, Hungary.
[2] Laboratory of Structural Chemistry and Biology ELTE Eötvös Loránd University, Pázmány Péter sétány 1/A, H-1117 Budapest, Hungary.
[3] Hevesy György PhD School of Chemistry, ELTE Eötvös Loránd University, Pázmány Péter sétány 1/A, H-1117 Budapest, Hungary.
[4] Laboratory of Interfaces and Nanostructures, Institute of Chemistry, Eötvös Loránd University, Pázmány Péter sétány 1/A, H-1117 Budapest, Hungary
[5] Department of Pharmaceutical Chemistry, Semmelweis University, Hőgyes Endre utca 9, 1092 Budapest, Hungary

a	Human/Hamster/Rat/Mouse/Camel/Dog/Pig:	HSQGTFTSDY SKYLDSRRAQ DFVQWLMNT
	Guinea Pig:	HSQGTFTSDY SKYLDSRRAQ QFLKWLLNV
	Degu:	HSQGTFTSDY SKFLDTRRAQ DFLDWLKNT
	Opossum:	HSQGTFTSDY SKYLDSRRAQ DFVQWLMST
	Chicken/Turkey:	HSQGTFTSDY SKYLDSRRAQ DFVQWLMST
	Turtles/Alligators/Duck:	HSQGTFTSDY SKYLDTRRAQ DFVQWLMST
	Gila monster lizard:	HSQGTFTSDY SKYLDTRRAQ DFVQWLMNT
	Salamander:	HSQGTFTSDY SKYLDNRRAQ DFIQWLMST
	Rana catesbeiana & tigrina:	HSQGTFTSDY SKYLDSRRAQ DFVQWLMNS
	Western clawed frog:	HSQGTFTSDY SKYLDSRRAQ DFIQWLMNT
	Alligator gar:	HSQGTFTNDY SKYLDTRRAQ DFVQWLMST
	Catfish:	HSEGTFSNDY SKYLETRRAQ DFVQWLMNS
	Goldfish:	HSEGTFSNDY SKYLETRRAQ DFVEWLMNS
	Anglerfish-1:	HSEGTFSNDY SKYLEDRKAQ EFVRWLMNN
	Anglerfish-2:	HSEGTFSNDY SKYLETRRAQ DFVQWLKNS
	Paddlefish-1:	HSQGMFTNDY SKYLEEKRAK EFVEWLKNGKS
	Paddlefish-2:	HSQGMFTNDY SKYLEEKSAK EFVEWLKNGKS
	Rainbow trout-1:	HSQEGTFSNY SKYQEERMAQ DFVQWLMNS
	Rainbow trout-2:	QSQEGTFSYY SKYQEERMAR DFLHWLMNS
	Sea lamprey-1:	HSEGTFTSDY SKYLENKQAK DFVRWLMNA
	Sea lamprey-2:	HSQGSFTSDY SKHLDVQKAK DFVTWLLNT
	River lamprey:	HSQGSFTSDY SKHLDVKQAK DFVIWLLNT
b	Human/Chimpanzee/Crab.eating macaque:	YAEGTFISDY SIAMDKIHQQ DFVNWLLAQK GKKNDWKHNI TQ
	Dog/Cat/Tiger/Horse/Chinese tree shrew:	YAEGTFISDY SIAMDKIRQQ DFVNWLLAQK GKKNDWKHNI TQ
	Pig:	YAEGTFISDY SIAMDKIRQQ DFVNWLLAQK GKKSDWKHNI TQ
	Rat/Armadillo/Europen shrew:	YAEGTFISDY SIAMDKIRQQ DFVNWLLAQK GKKNDWKHNL TQ
	Mouse:	YAEGTFISDY SIAMDKIRQQ DFVNWLLAQR GKKSDWKHNI TQ
	Brandt's bat/Little brown bat/*Myotis davdii*:	YAEGTFISDY SIAMDKIRQQ DFVDWLLAQK GKKNDWKHNI TQ
	Cape golden mole:	YAEGTFISDY SITMDKIRQQ DFVNWLLAQK GKKNDWKHNI TQ
	Camel:	YAEGTFISDY SIAMDKIRQQ DFVNWLLAQK GKKGDWKHNI TQ
	Cape elephant shrew:	YAEGTFISDY SIAMDKIRQQ DFVNWLLAQR GKKNDWKHNI TQ
	Hamster:	YAEGTFISDY SIALDKIRQQ DFVNWLLAQK GKKNDWKHNI TQ
	Florida mantee:	YAEGTFISDY SIAMDKIRQQ DFVNWLLAQK GKKKDWKHNI TQ
	Small madagascar hedgehog:	YAEGTFISDY SIAMDKIRQQ DFVNWLLAQK GKKNDWKHNM TQ
	Cattle:	YAEGTFISDY SIAMDKIRQQ DFVNWLLAQK GKKSDWIHNI TQ
	Star-nosled mole:	YAEGTFISDY SIAMDKIRQQ DFVNWLLAQK GKKSSWKHNL TQ
	Chicken:	YSEATLASDY SRTMDNMLKK NFVEWLLARR EKKSDNVIEP Y
	Mallard:	YSEATLASDY SRTMDNMLKK NFVEWLLARR EKKSDNVIET Y
	Budgerigar:	YSEATLASDY SRTMDNMLKK NFVEWLLARR EKKSDNMIEE Y
	Rock Pigeon:	YSEATLASDY SRTMDNMLKK NFVEWLLARR EKKSDNIEEP Y
	Saker falcon:	YSEATLASDY SRTMDNMLKK NFVEWLLARR EKKSDSAAEL R
	Tibetan ground-tit:	YSEATLASDY SRTMDHVLGK NFVEWLLARR ERKSQDVAEP L
	Sea turtle:	YSEAILASDY SRTMDNMLKK NFVEWLLARR EKKSENAIDP S
	Soft-shelled turtle:	YSEAILASDY SRTMDNMLKK NFVEWLLARR EKKSENDIDP S
	Western painted turtle:	YSEAILASDY SRTMDNMLKK NFVEWLLARR EKKSDNAIDP S
	American alligator:	YSEAILASDY SRTIDNMLKK NFVEWLLARR EMKMDNAIDP Y
	Western clawled frog:	YSEAILASDY SRSVDNMLKK NFVDWLLARR EKKSENTSEA T
	African clawed frog-1:	YSEAILASDY SRSVDNMLKK NFVDWLLARR EKKSDNTSEA T
	African clawed frog-2:	YSEAILASDY SRSVDNMLKK NFVDWLLARR EKKSENTSKA T
	Atlantic salmon:	YAESTIASDM SKIMDSMVQK NFVNFLLNQK E
	Zebrafish:	YAESTIASDI SKIVDSMVQK NFVNFLLNQR E
c	Human/Hamster/Rat/Mouse/Camel/Dog/Pig/Guinea Pig/Degu:	HAEGTFTSDV SSYLEGQAAK EFIAWLVKGR G
	Squirrel:	HAEGTFTSDV SSYLEGQAAK EFIAWLVKGR E
	Platypus:	HSEGTFTNDV TRLLEEKATV EFIAWLLKGL E
	Chicken/Turkey/Turtles/American alligator:	HAEGTYTSDI TSYLEGQAAK EFIAWLVNGR G
	Chinese alligator:	HAEGTYTSDI TSYLEGQAAK EFIAWLVNGR R
	Gila monster lizard:	HADGTYTSDI SSYLEGQAAK EFIAWLVNGR G
	Salamander:	HADGTLTSDI SSYLEGQAAK EFIAWLVNGR G
	Rana catesbeiana:	HADGTFTSDM SSYLEEKAAK EFVDWLIKGR P
	Rana tigrina-2:	HADGTFTSDM TSYLEEKAAK EFVDWLIKGR P
	Rana tigrina-1:	HADGTYTNDV TQFLEEKAAK EFVDWLIKGK P
	Alligator gar:	HADGTYTSDV SSYLQDQAAK KFVTWLKQGQ D
	Catfish:	HADGTYTSDV SSYLQDQAAK DFITWLKSGQ P
	Anglerfish-1:	HADGTFTSDV SSYLKDQAIK DFVDRLKAGQ V
	Anglerfish-2:	HADGTYTSDV SSYLQDQAAK DFVSWLKAGR G
	Paddlefish:	HADGTYTSDA SSFLQEQAAR DFISWLKKGQ
	Rainbow trout-1:	HADGTYTSDV STYLQDQAAK DFVSWLKSGP A
	Rainbow trout-2:	HADGTYTSDV STYLQKQAAK DFVSWLKSGR A
	Sea lamprey:	HADGTFTNDM TSYLDAKAAR DFVSWLARSD KS
d	Human:	HADGSFSDEM NTILDNLAAR DFINWLIQTK ITD
	Mouse:	HADGSFSDEM STILDNLAAR DFINWLIQTK ITD
	Hamster/Rat/Guinea pig:	HADGSFSDEM NTILDSLATR DFINWLIQTK ITD
	Camel:	HADGSFSDEM NTILDNLATQ DFINWLLQTK ITD
	Dog:	HADGSFSDEM NTVLDTLATR DFINWLLQTK ITD
	Degu:	HADGSFSDEM NTVLDHLATK DFINWLIQTK ITD
	Pig:	HADGSFSKEM NTVLDNLATR DFINWLLHTK ITDSL
	Chicken/turekey/Alligators/Painted turtle:	HADGTFTSDI NKILDDMAAK EFLKWLINTK VTQ
	Soft-shelled turtle:	HADGTFTSDF NKILDDMAAK EFLKWLINTK VTQ
	Gila monster lizard:	HADGTFTSDY NQLLDDIATQ EFLKWLINQK VTQ
	Rana catesbeiana & tigrina:	HADGSFTSDF NKALDIKAAQ EFLDWIINTP VKE
	Western clawled frog:	HADGSFTNDI NKVLDIIAAQ EFLDWVINTQ VTE
	Salamander:	HADGSFTSDI NKVLDTIAAK EFLNWLISTQ VTE
	Rainbow trout-1/2:	HVDGSFTSDV NKVLDSLAAK EYLLWVMTSK TSG
	Sea lamprey-1:	HAEDVMALIL RTMAKTDFEN WEKQNSNTQT D
	Sea lamprey-2:	HSDGSFTNDM NVMILRMSAK NFLEWLKQQG RG

Supplementary Figure 1: Comparison of the amino acid sequences of a) glucagon, b) GIP, c) GLP-1, and d) GLP-2 in vertebrates. Residues of the conserved xFxxWL motifs of the common aggregation cores are shaded grey. Sequence comparisons adapted from the Handbook of Hormones[1] and based on figures Fig.26c.1, Fig.26d.1, Fig.26b.1 and Fig.26a.1.

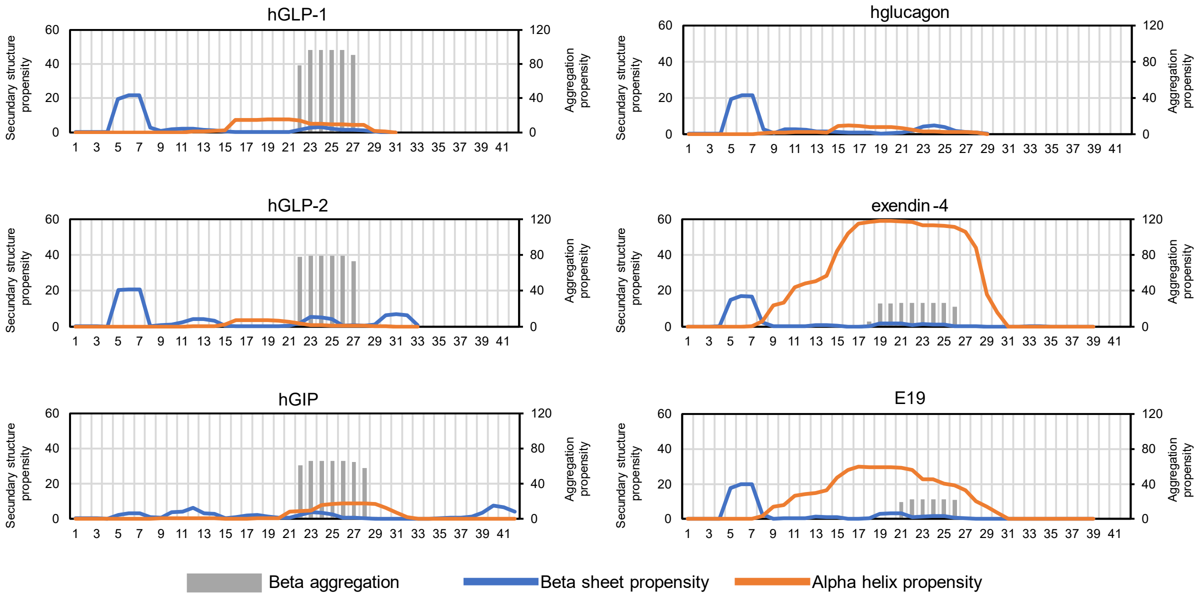

Supplementary Figure 2: The TANGO[2] algorithm was used to predict the secondary structure and aggregation propensity of the full-length hormone peptides. The settings used were *pH* 7, temperature of 298.15 K, ionic strength of 0.02 M, and a peptide concentration of 1 M. The algorithm predicts that the same site in all sequences is prone to aggregation but to different degrees.

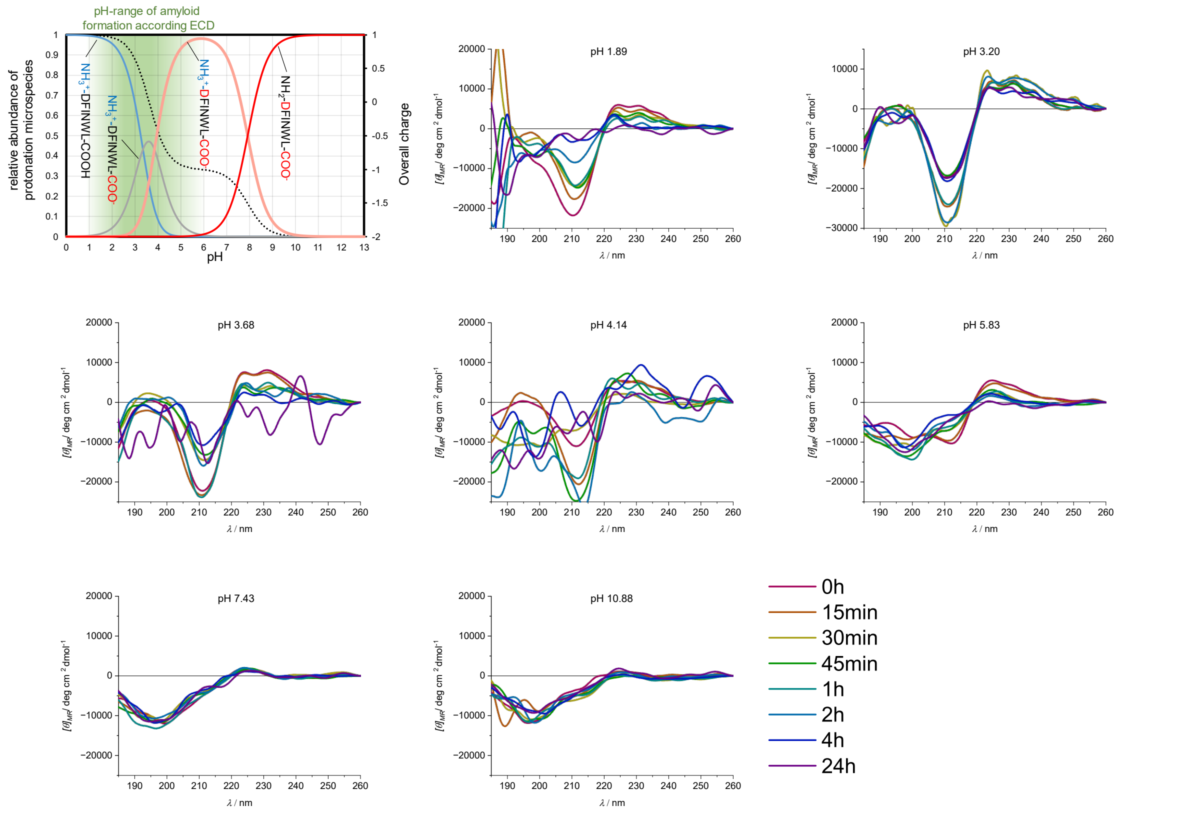

Supplementary Figure 3: The pH-dependent distribution of microspecies and total charge of APR^{gluc}, along with a set of pH and time-dependent far-UV CD spectra. (The applied pK_a values can be found in **Supplementary Table 4**.)

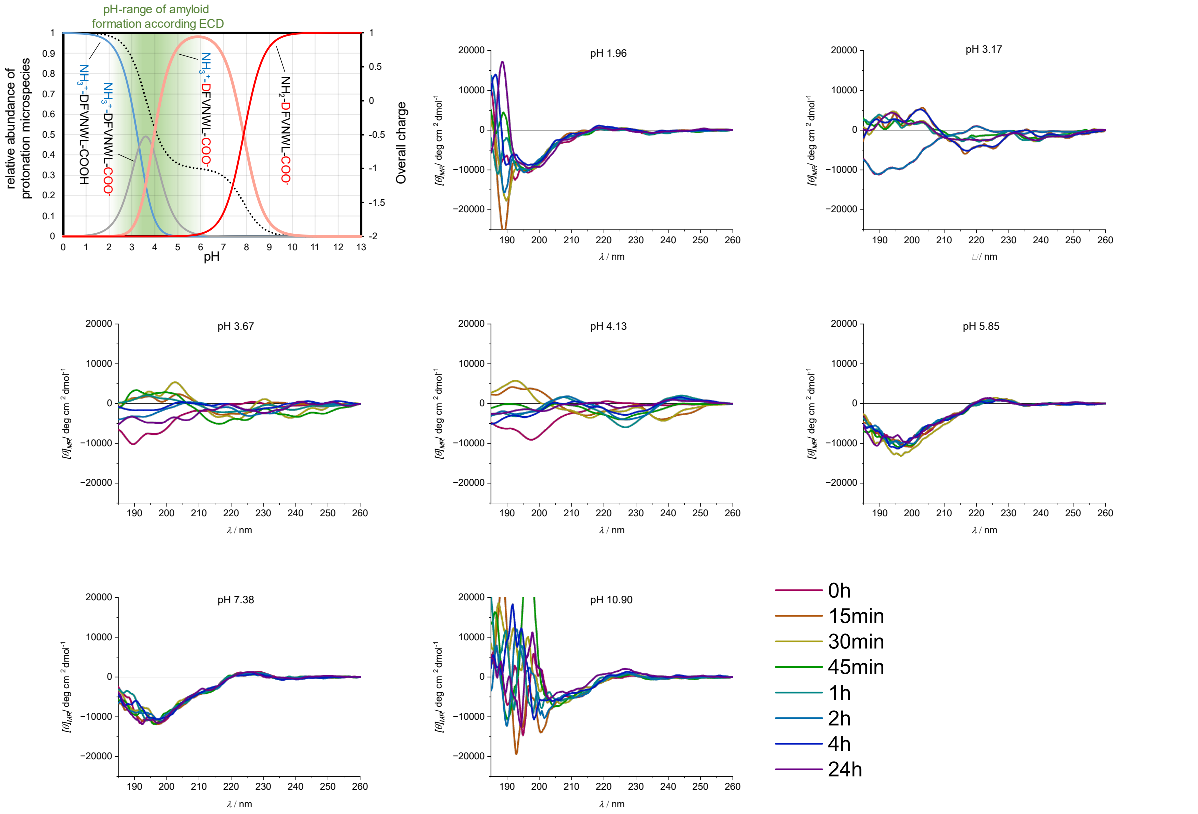

Supplementary Figure 4: The pH-dependent distribution of microspecies and total charge of APR^{GIP}, along with a set of pH and time-dependent far-UV CD spectra. (The applied pK_a values can be found in **Supplementary Table 4**.)

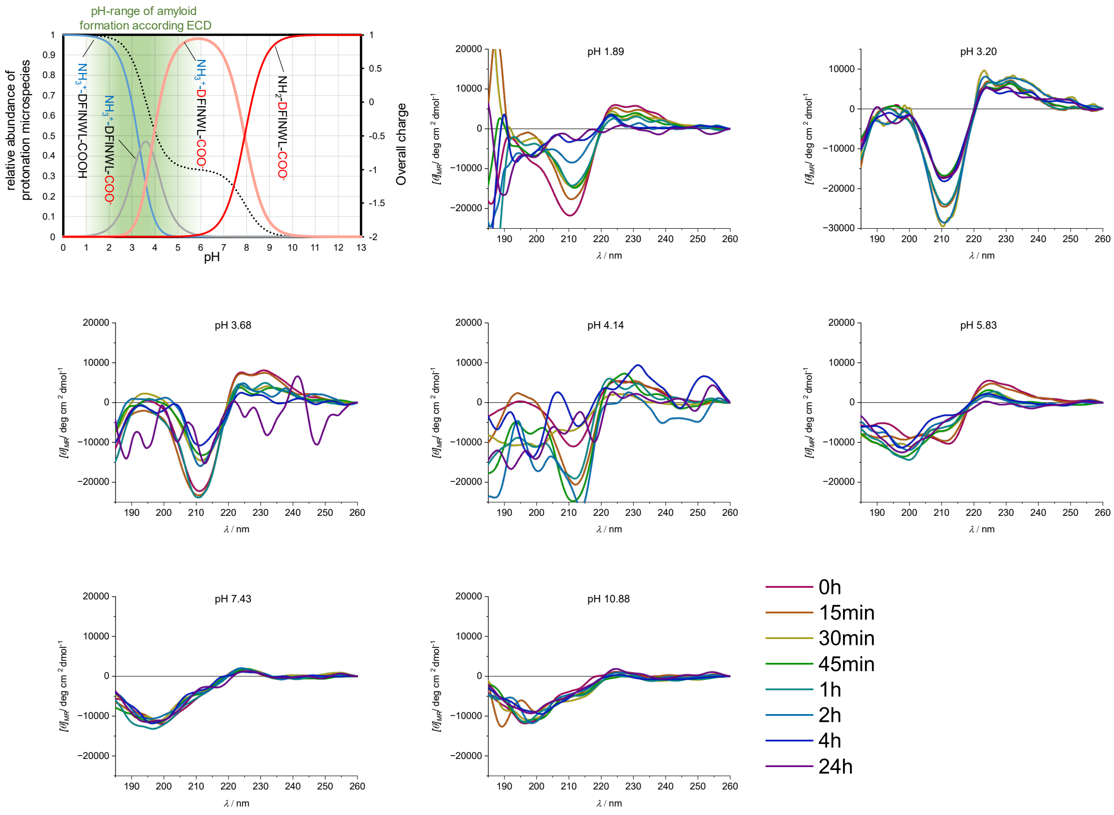

Supplementary Figure 5: The pH-dependent distribution of microspecies and total charge of APR^{GLP2}, along with a set of pH and time-dependent far-UV CD spectra. (The applied pK_a values can be found in **Supplementary Table 4**.)

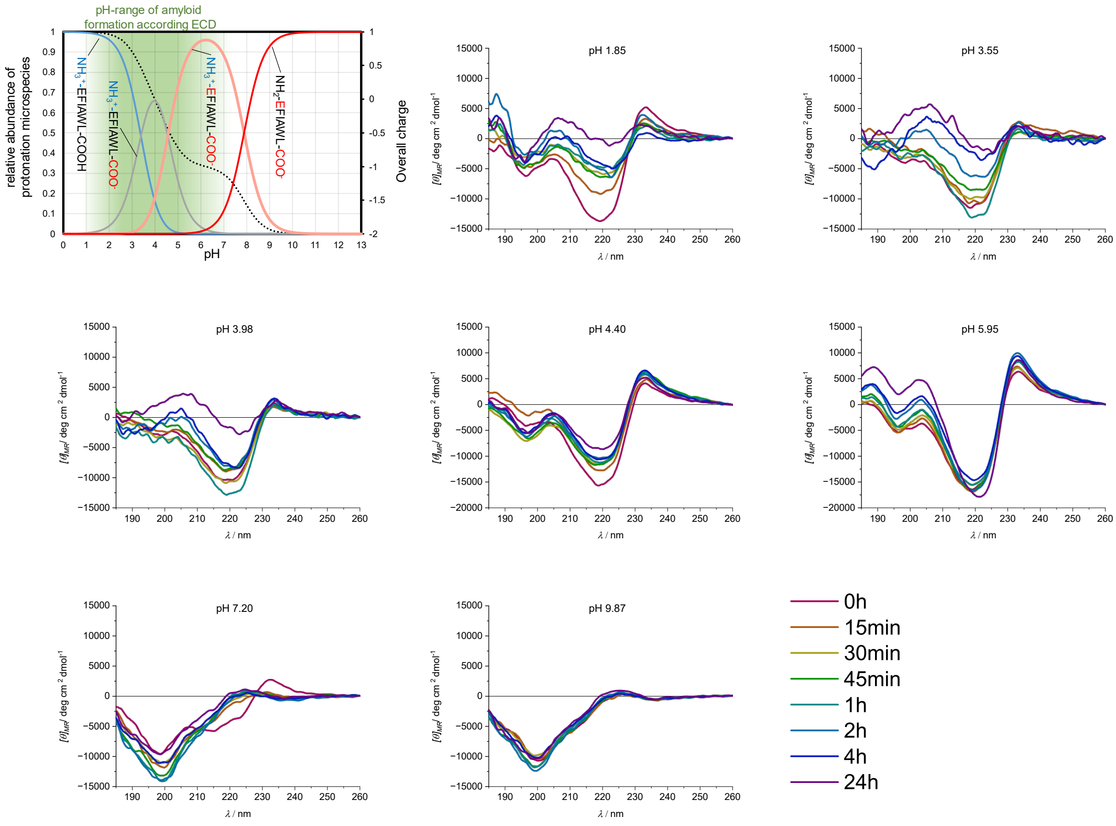

Supplementary Figure 6: The pH-dependent distribution of microspecies and total charge of APR^{GLP1}, along with a set of pH and time-dependent far-UV CD spectra. (The applied pK_a values can be found in **Supplementary Table 4**.)

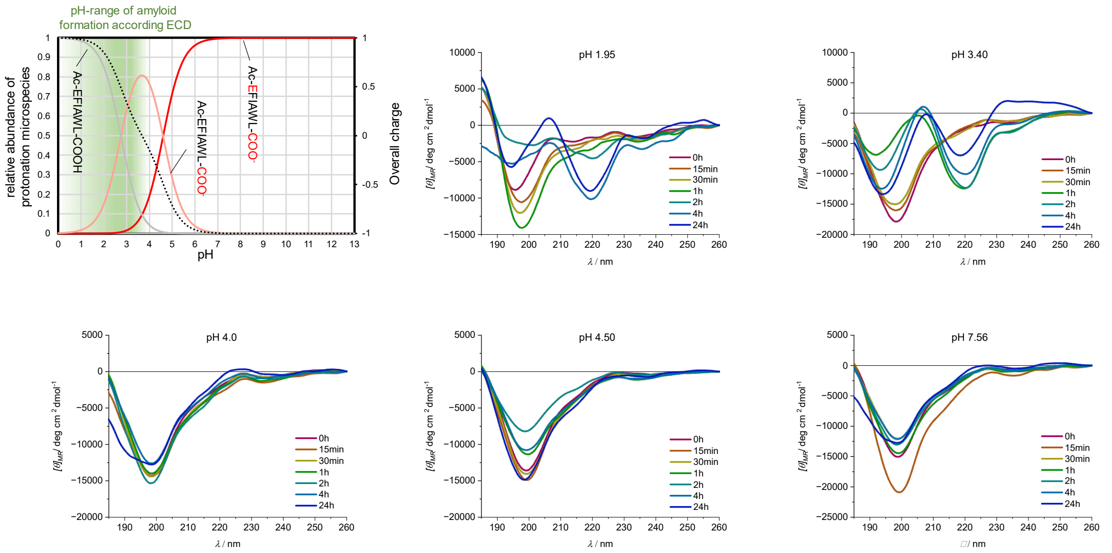

Supplementary Figure 7: The pH-dependent distribution of microspecies and total charge of APR^{GLP1} – Ac-EFIAWL, along with a set of pH and time-dependent far-UV CD spectra. (The applied pK_a values can be found in **Supplementary Table 4**.)

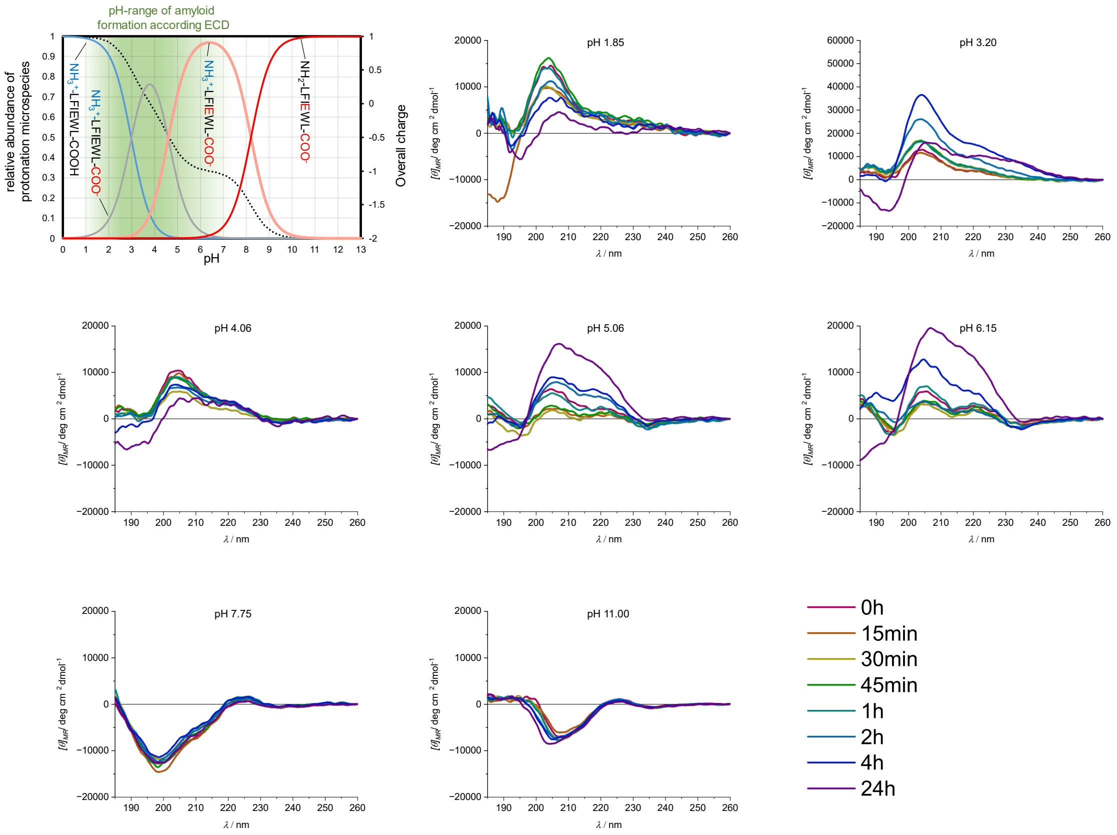

Supplementary Figure 8: The pH-dependent distribution of microspecies and total charge of APR$^{ex\text{-}4}$, along with a set of pH and time-dependent far-UV CD spectra. (The applied pK_a values can be found in **Supplementary Table 4**.)

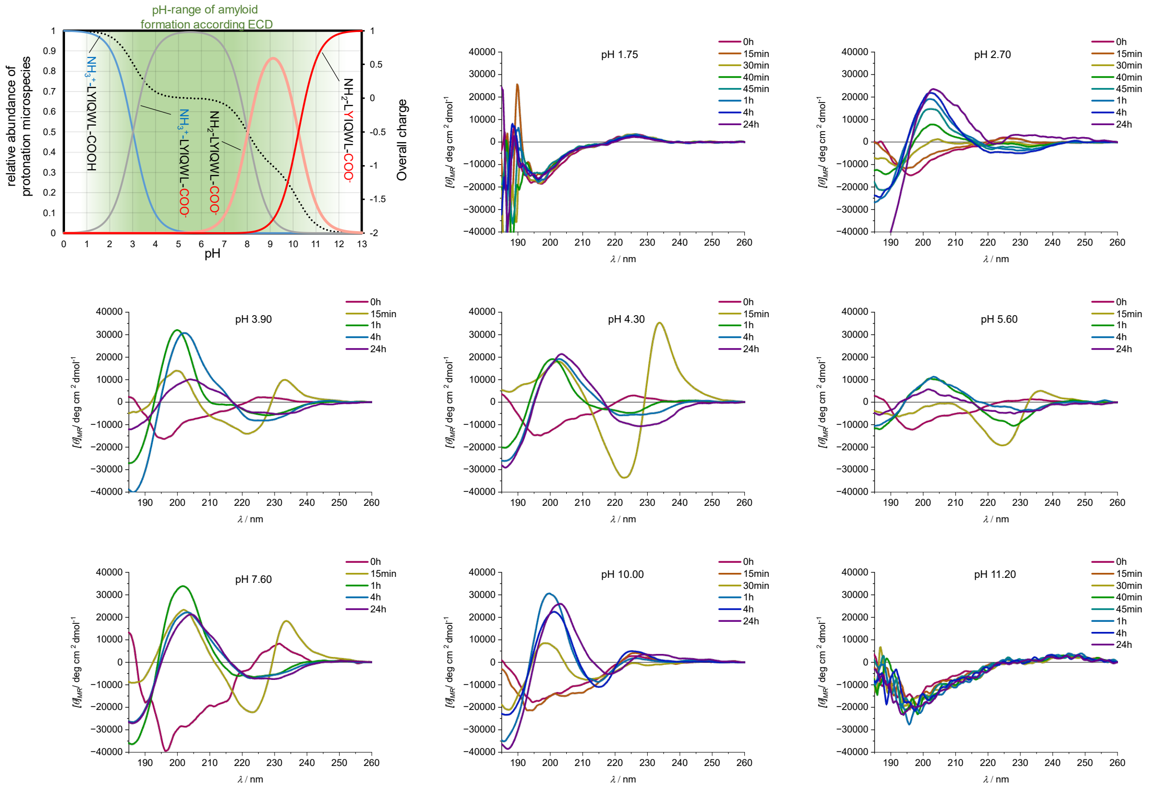

Supplementary Figure 9: The pH-dependent distribution of microspecies and total charge of APR^{Tc5b}, along with a set of pH and time-dependent far-UV CD spectra. (The applied pK_a values can be found in **Supplementary Table 4**.) Note, the initial amyloid nucleation is dominated by transient signals (T), which are represented by the dark yellow – at 15 minutes - on the chart between a pH range of 3.9 – 7.6.

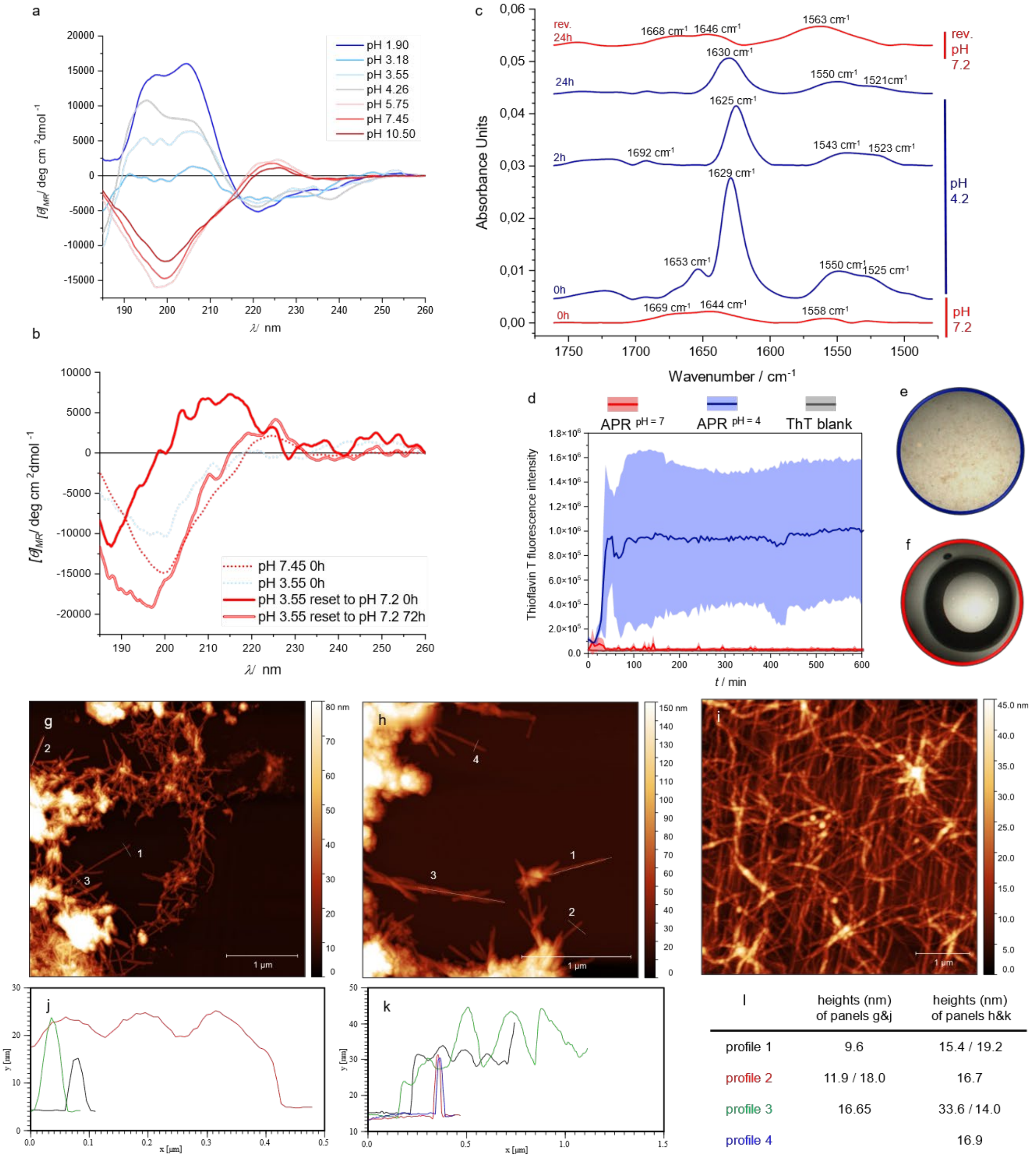

l	heights (nm) of panels g&j	heights (nm) of panels h&k
profile 1	9.6	15.4 / 19.2
profile 2	11.9 / 18.0	16.7
profile 3	16.65	33.6 / 14.0
profile 4		16.9

Supplementary Figure 10: Amyloid formation of APR^{gluc} - DFVQWL characterized by several techniques: **a)** CD spectra measured after 4 h of stirring at 37°C at different pHs. **b)** APR hexapeptide reversibility followed by CD. The initial (t = 0h) spectra are shown by dotted lines at acidic (light blue) and neutral pH (red). After one week of stirring at 37°C, the pH of the mature aggregated sample was adjusted from *pH* 3.55 to 7.2 and the CD spectrum was recorded immediately (red line). The spectrum was recorded again after stirring this pH-adjusted sample for 3 days at 37°C (red line doubled). The molar ellipticity values shown in panels **a)** and **b)** are corrected with the concentrations measured at the actual sampling points. **Supplementary Figure 3** shows all measured CD spectra. **c)** FTIR spectra (acid - blue, neutral - red) are stacked from bottom to top as a function of time. The appearance of the amide I band position (maximum at 1625 - 1630 cm^{-1}) immediately after pH adjustment to 4.2 shows the presence of an immediate secondary structure conversion towards β-sheets. The observed increase in absorbance compared to the native unfolded state indicates the intermolecular association of the individual β-strands into an extensive fibrillar structure. By adjusting the pH of the 24-hour aged sample to 7.2, the band associated with the fibrillar structure disappears and the original FTIR spectrum is restored. **d)** Real-time monitoring of Thioflavin T fluorescence intensity. The lines on the graph represent the average, while the lighter-shaded areas indicate the standard error, which was calculated from three parallel measurements. The ThT binding assay clearly demonstrates the amyloid

formation of APR^{gluc} under acidic conditions. Images were taken of wells containing both **e)** acidic and **f)** neutral samples after a 24-hour incubation period revealing visually observable extensive aggregation under acidic conditions. **g-i)** The morphology of fibrils in the 24-hour agitated acidic FTIR samples was characterized using AFM. Picture **i)** was taken from the optically dense region of the mica surface, while pictures g-h) were taken from an optically less dense area. (The brighter region in **Supplementary Figure 16/a**) Analysis of the cross-section height profiles (**j-l**) reveals that individual (proto)filaments are twisting together to form larger fibrils. (**j/g** – profile 2, **h/a** – profile 1 and 3).

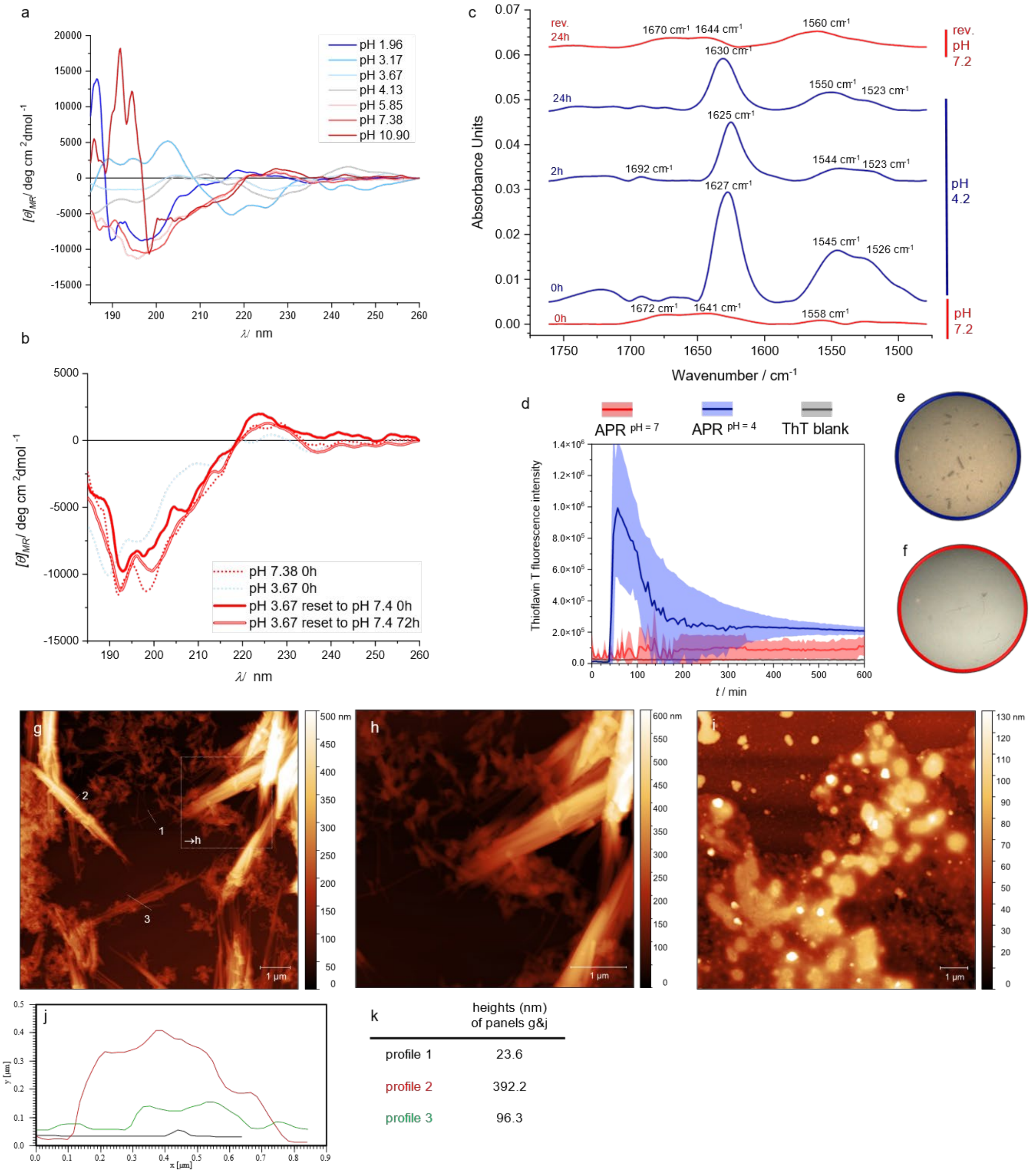

Supplementary Figure 11: Amyloid formation of APR^{GIP} - DFVNWL: **a)** CD spectra measured after 4 h of stirring at 37°C at different pHs. **b)** APR hexapeptide reversibility followed by CD. The initial (t = 0h) spectra are shown by dotted lines at acidic (light blue) and neutral pH (red). After one week of agitation at 37°C, the pH of the mature aggregated sample was adjusted from *pH* 3.67 to 7.38 and the CD spectrum was immediately recorded (red line). This pH-adjusted sample was stirred further at 37°C for 3 days (red line doubled). The molar ellipticity values shown in panels **a)** and **b)** are corrected with the concentrations measured at the actual sampling points. **Supplementary Figure 4** shows all measured CD spectra. **c)** FTIR spectra (acid - blue, neutral - red) are stacked from bottom to top as a function of time. After adjusting the pH to 4.2, the position of the emerging amide I band shows a maximum peak at 1625 - 1630 cm^{-1}, indicating an immediate conversion of

the secondary structure to β-sheets. The band associated with the fibrillar structure disappears and the initial FTIR spectrum is restored in the 24 h matured sample after pH adjustment to 7.2. **d)** Real-time monitoring of thioflavin T fluorescence intensity. The lines on the graph represent the average, while the lighter-shaded areas indicate the standard error calculated from three parallel measurements. The sharp increase in ThT fluorescence within the first hour demonstrates the amyloid formation of APR[gluc] under acidic conditions. Photographs of wells containing both **e)** acidic and **f)** neutral samples after 24 hours of incubation show extensive aggregation under acidic conditions. **g-h)** The morphology of the fibrils in the acidic FTIR samples stirred for 24 h was characterized by AFM. Cross-sectional analysis **j-k)** shows the diameters of the fibrils formed from the smallest detectable protofilaments to the bundled fibrils. **i)** When we vacuum-dried the pH-adjusted FTIR sample (c-panel, top spectrum - rev.pH 7.2) and examined the surface of the mica (**Supplementary Figure 16/b**), we observed a molecular layer covering the surface instead of the fibrillar aggregates seen earlier, providing visual evidence for the reversibility of amyloid formation.

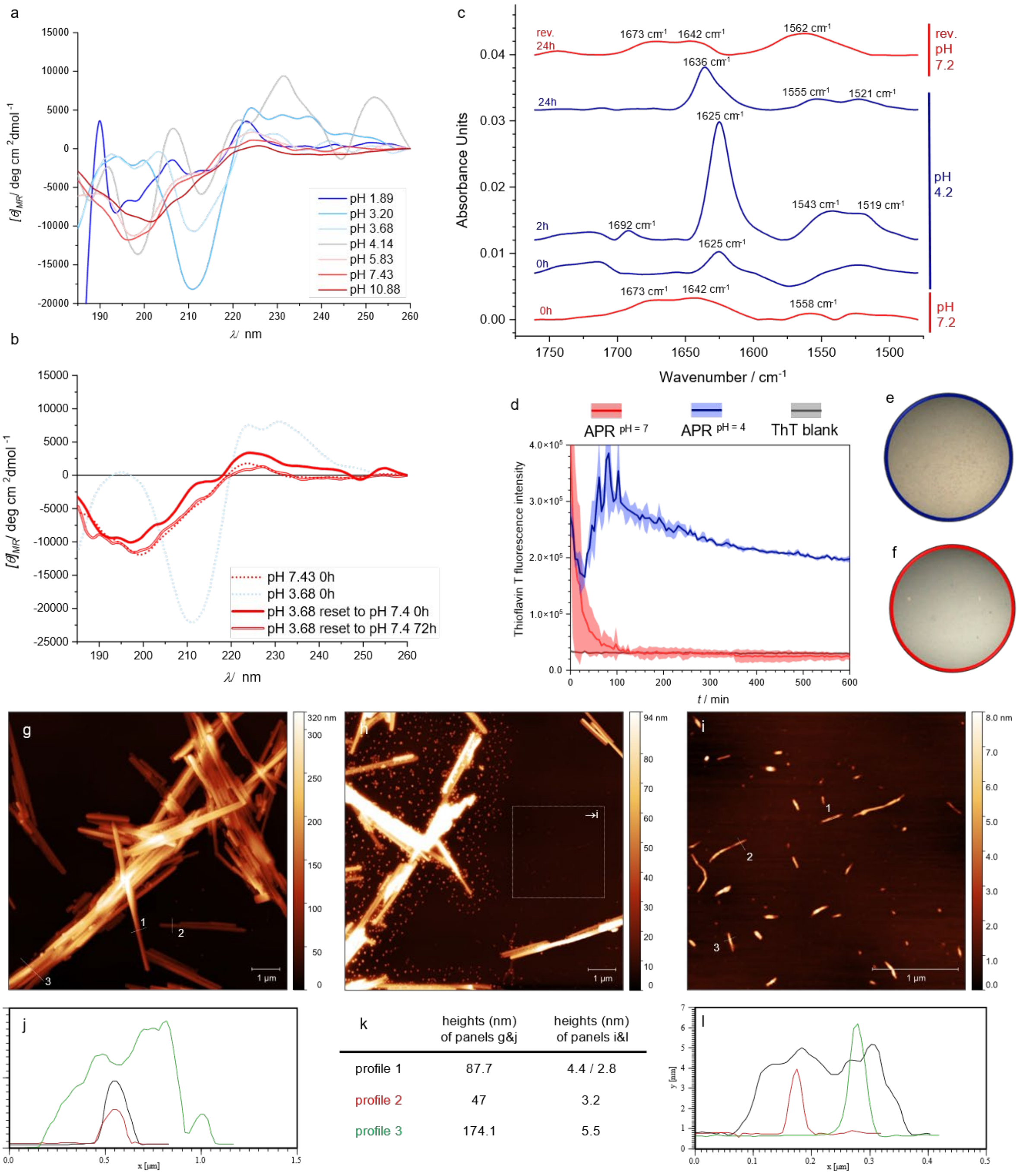

	heights (nm) of panels g&j	heights (nm) of panels i&l
profile 1	87.7	4.4 / 2.8
profile 2	47	3.2
profile 3	174.1	5.5

Supplementary Figure 12: Amyloid formation of APR[GLP2] - DFINWL: **a)** CD spectra measured after 4 h of stirring at 37°C at different pHs. **b)** APR hexapeptide reversibility followed by CD. The initial (t = 0h) spectra are shown by dotted lines at acidic (light blue) and neutral pH (red). After one week of stirring at 37°C, the pH of the mature aggregated sample was adjusted from 3.68 to 7.4. The CD spectrum was recorded immediately after the pH adjustment (red line) and the pH-adjusted sample

was stirred for 3 days at 37°C (doubled red line). The molar ellipticity values in panels **a)** and **b)** were corrected on the basis of the concentrations measured at each sampling point. **Supplementary Figure 5** shows all measured CD spectra. **c)** FTIR spectra (acid - blue, neutral - red) are stacked from bottom to top as a function of time. After adjusting the pH to 4.2, the emerging amide I band position shows a maximum peak at 1625 - 1630 cm^{-1}, indicating the conversion of the secondary structure to β-sheets after 2 hours. After adjusting the pH to 7.2, the band associated with the fibrillar structure disappears and the FTIR spectrum of the 24-hour matured sample returns to its initial state. **d)** Real-time monitoring of thioflavin T fluorescence intensity. The lines on the graph represent the average values, while the lighter-shaded areas indicate the standard error calculated from three parallel measurements. The increase in measured ThT fluorescence in the acidic sample (blue) is less pronounced compared to the intensity of the other APRs examined here. Nevertheless, the peak is about 10 times more intense than that of the pH 7 sample (red), which is considered to be ThT negative. Interestingly, the latter sample shows an initial ThT fluorescence which decays to the level of the blank ThT intensity within the first two hours. It is possible that undissolved aggregates remained from the sample preparation process, although it was carried out thoroughly. Images of wells containing both **e)** acidic and **f)** neutral samples after 24 h incubation showed visually observable extensive aggregation under acidic conditions. **g-l)** Fibril morphology of the 24 h stirred acidic FTIR samples characterized by AFM. Panel **i)** shows the size of the smallest detectable protofilaments (3 - 6 nm in height), which already show periodicity in their morphology. Initially flexible, the protofilaments assemble into extensive nanostructures, gradually becoming more linear and rigid.

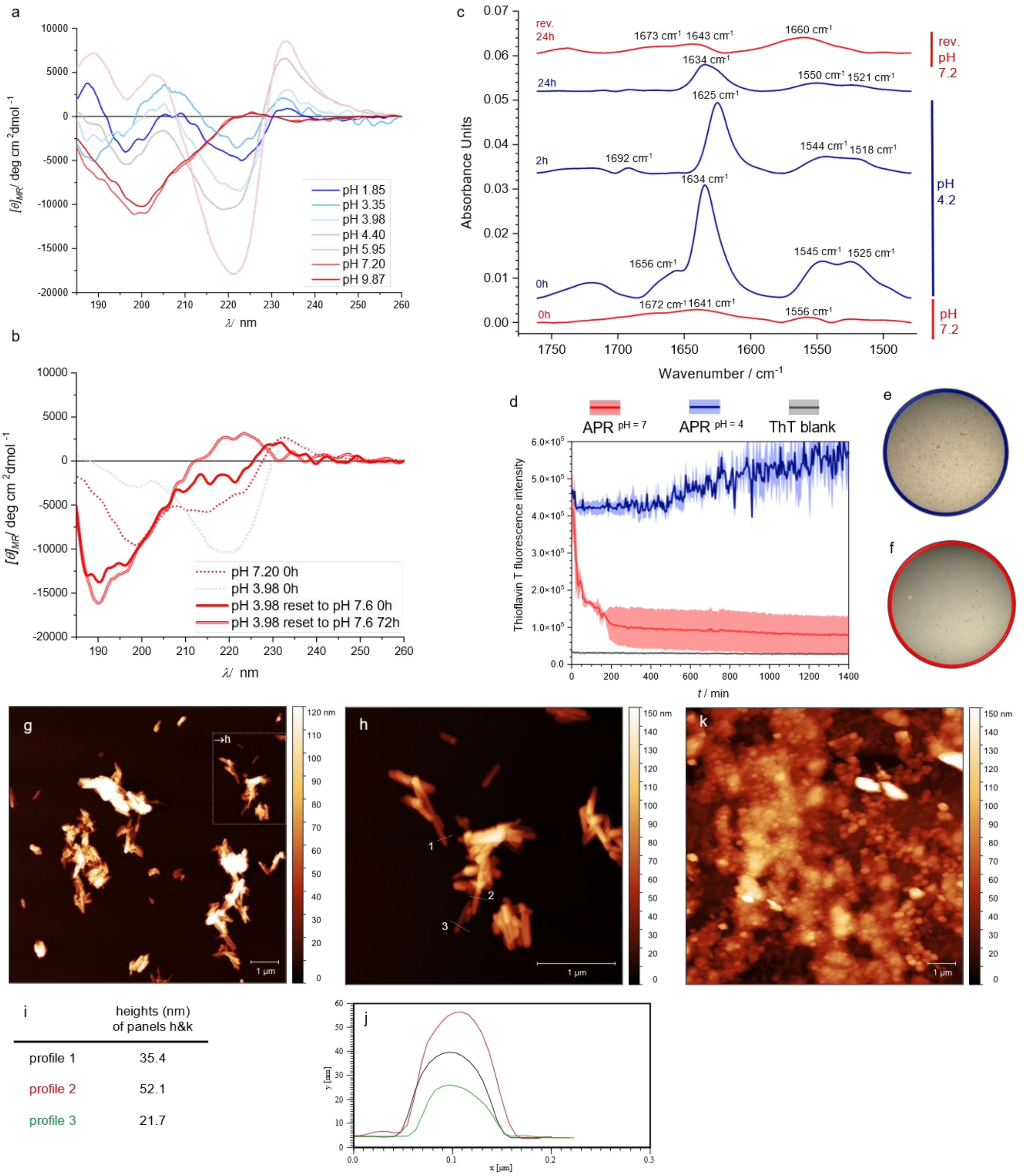

Supplementary Figure 13: Amyloid formation of APR^{GLP1}-EFIAWL: **a)** CD spectra measured after 4 h of stirring at 37°C at different pHs. **b)** APR hexapeptide reversibility followed by CD. The initial (*t* = 0h) spectra are shown by dotted lines at acidic (light blue) and neutral pH (red). After one week of stirring at 37°C, the pH of the mature aggregated sample was adjusted from 3.98 to 7.6. The CD spectrum was recorded immediately after the pH adjustment (red line) and the pH-adjusted sample was stirred for 3 days at 37°C (doubled red line). The molar ellipticity values in panels a) and b) were corrected on the basis of the concentrations measured at each sampling point. **Supplementary Figure 6** shows all measured CD spectra. **c)** FTIR spectra (acid - blue, neutral - red) are stacked from bottom to top as a function of time. After adjusting the pH to 4.2, the emerging amide I band position shows a maximum peak at 1625 - 1635 cm^{-1}, indicating the conversion of the secondary structure to β-sheets. After adjusting the pH to 7.2, the band associated with the fibrillar structure disappears, and the FTIR spectrum of the 24-hour matured sample returns to its initial state. **d)** Thioflavin T fluorescence intensity monitored in real-time. The lines on the graph represent the average values, while the lighter-shaded areas indicate the standard error calculated from three parallel measurements. The increase in ThT fluorescence measured in the acidic sample (blue) is not as significant as in APR$^{ex\text{-}4}$. It also shows a rapid initial decay of ThT fluorescence similar to APR^{GLP2}. Visual inspection of images taken from wells containing both acidic **e)** and neutral **f)** samples after 24 hours of incubation revealed aggregation only under acidic conditions. **g-j)** Fibril morphology of the 24 h stirred acidic FTIR samples characterized by AFM. Panel **k)** shows

that the previously formed fibrillar nanostructures are no longer present in the mature FTIR sample after adjusting its pH to 7.2. In addition, the redissolved APR forms a layer of non-fibrillar aggregates on the substrate.

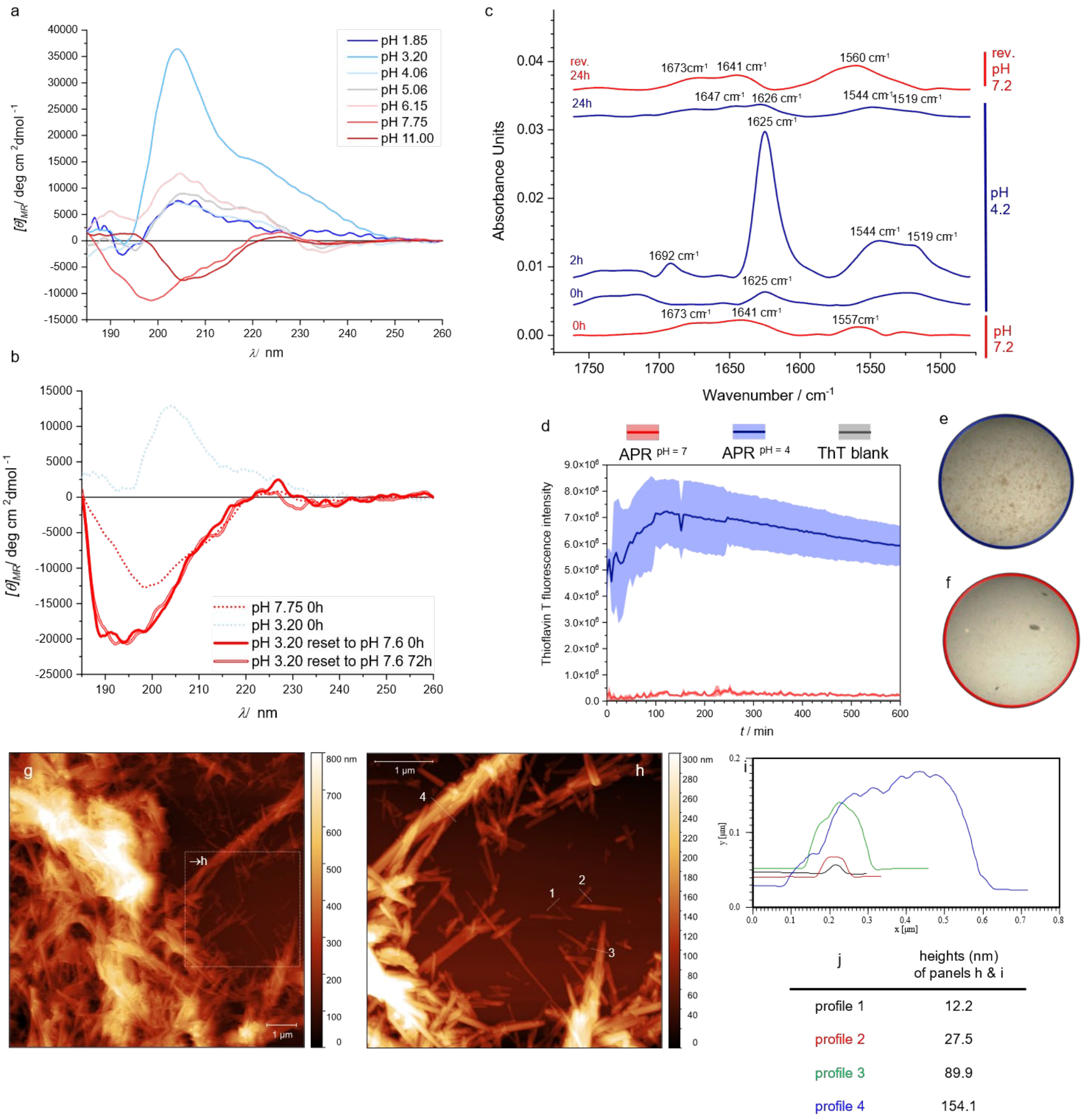

Supplementary Figure 14: Amyloid formation of APR^{ex-4}- LFIEWL: **a)** CD spectra measured after 4 h of stirring at 37°C at different pHs. **b)** APR hexapeptide reversibility followed by CD. The initial (t = 0h) spectra are shown by dotted lines at acidic (light blue) and neutral pH (red). After one week of stirring at 37°C, the pH of the mature aggregated sample was adjusted from 3.20 to 7.6. The CD spectrum was recorded immediately (red line), then the sample was stirred for another 3 days at 37°C (red line doubled). The molar ellipticity values in panels a) and b) were corrected on the basis of the concentrations measured at each sampling point. **Supplementary Figure 8** shows all measured CD spectra. **c)** FTIR spectra (acid - blue, neutral - red) are stacked from bottom to top as a function of time. After adjusting the pH to 4.2, the emerging amide I band position shows a maximum peak at 1625 cm^{-1}, indicating the conversion of the secondary structure to β-sheets. By adjusting the pH of the 24 h matured sample to 7.2, the process can be reversed. **d)** Thioflavin T fluorescence intensity monitored in real-time. The lines on the graph represent the average values, while the lighter-shaded areas indicate the standard error calculated from three parallel measurements. The ThT binding assay clearly shows the amyloid formation of APR^{ex-4} under acidic conditions. After 24 hours of incubation, photographs of wells containing both acidic **e)** and neutral **f)** samples showed observable aggregation only in the acidic samples. **g-j)** Fibril morphology of the 24 hours stirred acidic FTIR samples characterized by AFM. Panel **h)** shows both the size of the small protofilaments and their increasing diameter as they assemble into larger nanostructures.

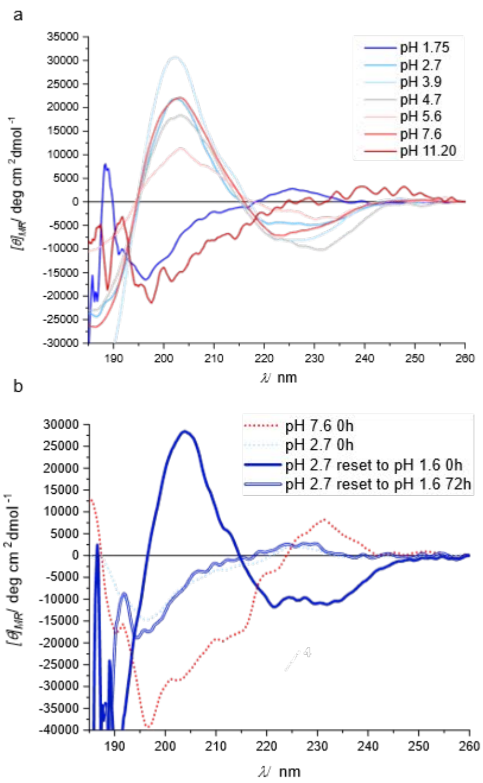

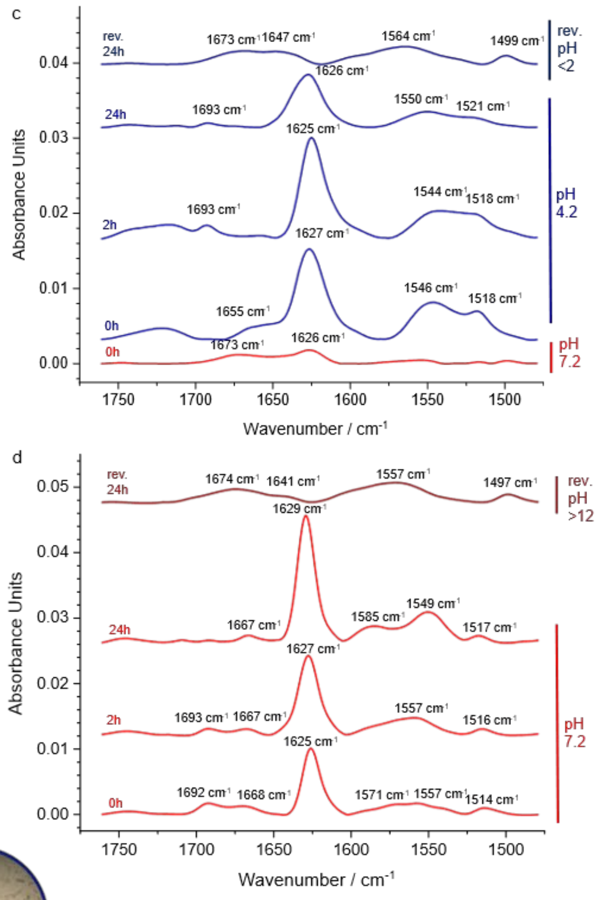

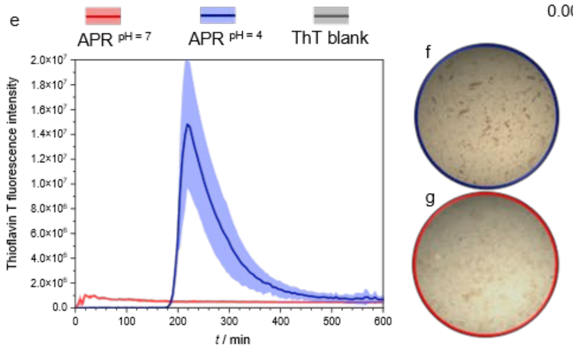

n	heights (nm) of panels i & j	heights (nm) of panels l & m
profile 1	10.3	11.6
profile 2	37.8	22.9
profile 3	84.9	38.8
profile 4	125.1	90.2

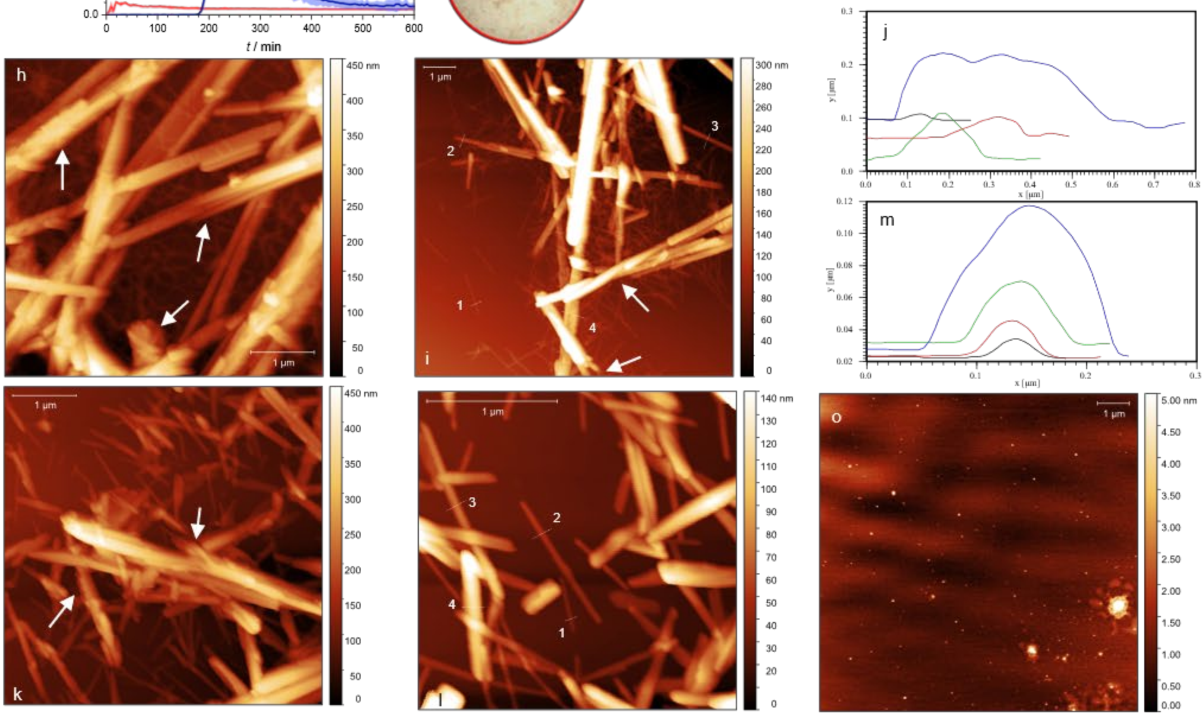

Supplementary Figure 15: Amyloid formation of APR^{Tc5b} - LYIQWL over an extended pH range: **a)** CD spectra measured after 4 h of stirring at 37°C at different pHs. **b)** APR hexapeptide reversibility followed by CD. The initial (*t* = 0h) spectra are shown by dotted lines at acidic (light blue) and neutral pH (red). After one week of incubation, the pH of the mature aggregated sample was adjusted from *pH* 2.7 to 1.6 and the CD spectrum was recorded immediately (blue line) and three days later (double blue line). The reversibility data suggest that the full recovery of the U-folded spectra takes more time compared to those where the side chain charge controls the on-off mechanism of aggregation. The molar ellipticity values are corrected with the concentrations measured at the actual sampling points**. Supplementary Figure 9** shows all the measured CD spectra. **c-d)** The FTIR spectra are arranged in chronological order from bottom to top, with neutral pH in red and acidic pH in blue (note that panel C and panel D show different initial *pH* 7.2 FTIR spectra as they were obtained from different samples). The amide I band peaking at 1625 - 1629 cm^{-1} indicates the presence of β-sheet aggregates at both *pH* 4.2 and 7.2. However, exposure of pretreated amyloid samples to extreme pH conditions (below 2 or above 12) led to the disappearance of these aggregates. Interestingly, we did not observe a delay in reversibility as in the case of CD measurements. **e)** Thioflavin T measurements confirm that APR^{tc5b} undergoes amyloid formation and shows the most intense fluorescence peak among the APRs studied in the present work. Interestingly, while the acidic form of APR^{tc5b} shows a high Thioflavin T intensity (619.9), its neutral form shows only a fraction of the fluorescence intensity (22.2), but is still comparable in magnitude to the Thioflavin T intensities of other acidic APRs such as APR^{GLP1} (18.5) and APR^{GLP2} (9.9). The lines on the graph represent the average values, while the lighter-shaded areas indicate the standard error calculated from three parallel measurements. Images were taken of wells containing both **f)** acidic and **g)** neutral samples after 24 hours of incubation showed visually observable aggregation. AFM revealed the fibril morphology of the 24-hour incubated **h-j)** neutral (*pH* 7.2) and **k-l)** acidic (*pH* 4.2) FTIR samples. Extensive fibril formation occurs under both conditions. The individual filaments detected have heights in the same range, around 10 nm (**j**/**m**/**n** profile 1). The white arrows indicate the entanglement of the fibrils, emphasizing their fibrillar nature rather than their nanocrystalline appearance. **o)** The AFM image of the FTIR sample taken after adjusting the pH to above 12 clearly showed that fibrillar particles were completely absent. Only a smooth molecular layer was observed.

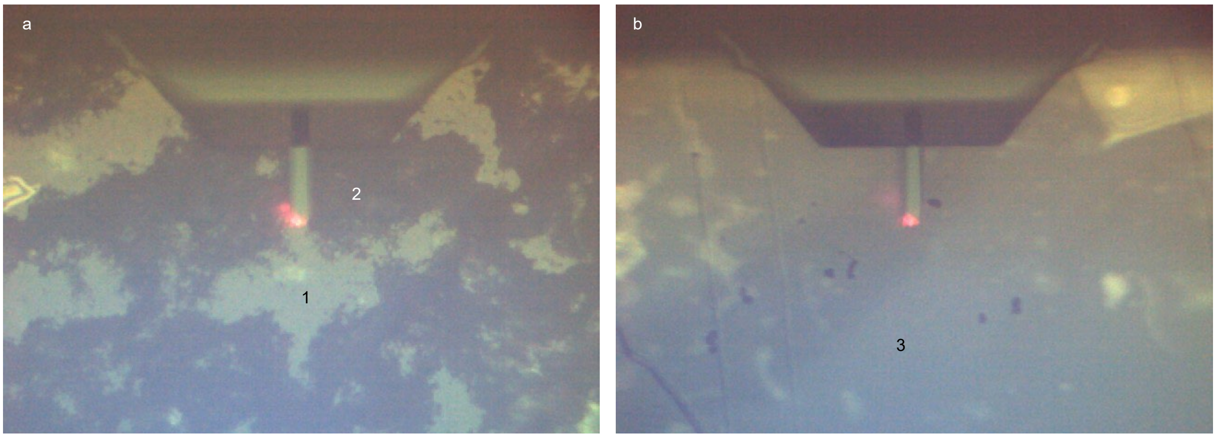

Supplementary Figure 16: Examples of different surface topologies of vacuum-dried samples on the mica surface are shown by images taken by the built-in AFM camera. **a)** Vacuum-dried sample of APR^{tc5b} stirred for 24 hours at neutral pH. Marker 1 indicates the clearer area where the base of the mica can be seen on the AFM images. Marker 2 indicates the optically dense area where the aggregated sample has sedimented. The majority of the recorded AFM images were taken from the boundary between these two areas. **b)** The vacuum-dried sample of the same APR^{tc5b} sample after pH readjustment (*pH* > 12) shows that most of the aggregated bulk has disintegrated to form a uniformly distributed layer over most of the surface. However, several patches of higher-density aggregates remained.

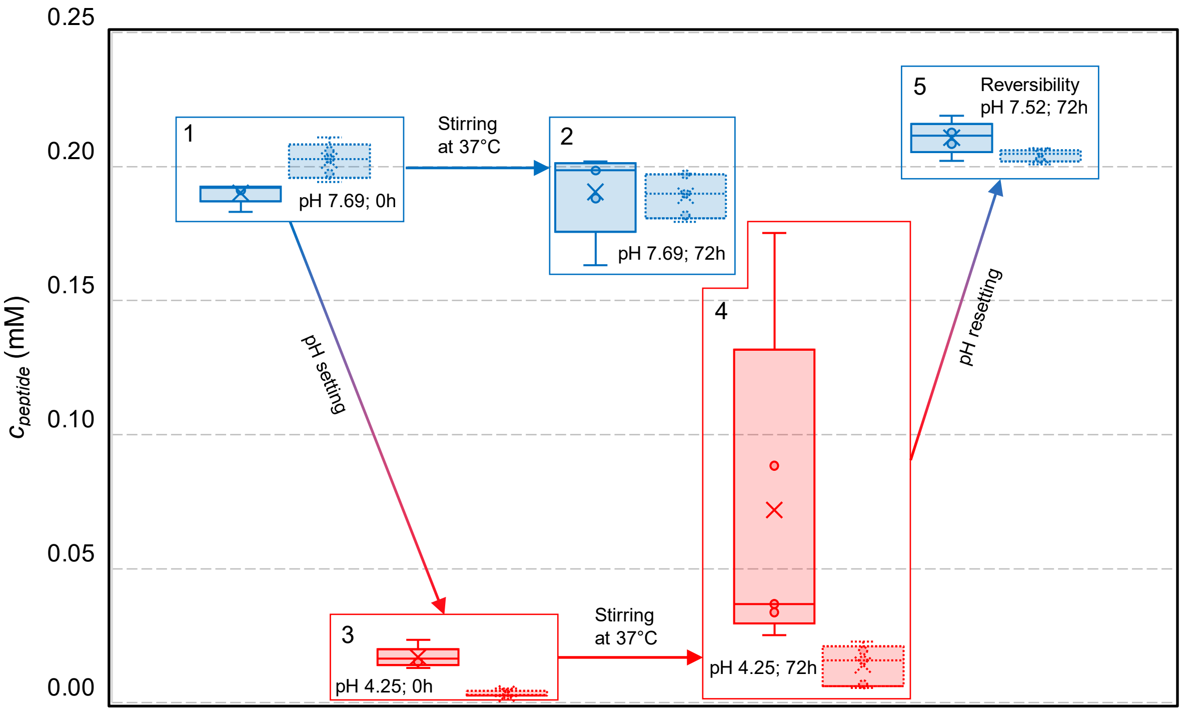

Supplementary Figure 17: Flowchart illustrating the Boxplot representation of APR^{GLP-1} solubility measured by Nanodrop. The box in the flowchart represents the interquartile range, with the lower bound indicating the first quartile and the upper bound indicating the third quartile. The whiskers in the plot extend to the minimum and maximum values, excluding any outliers. Individual data points displayed within the box represent the measured values. The "x" symbol represents the mean value of the data. The horizontal line inside the box represents the median value. The plain contoured boxes indicate the measurements of non-centrifuged samples, while the dotted boxes show the concentrations measured after centrifugation at 13000 RPM for 1 minute. The concentration of the sample was measured five times at each stage **(1-5)**. The concentration of the initial **(1)** and the same 72-hour agitated **(2)** sample was measured at neutral pH (blue). To test the effect of pH on solubility **(3)**, the pH was adjusted from neutral to acidic (red) after taking out half of the initial volume. The added volume (15 µl) of the 0.1 M HCl was insignificant compared to the whole volume (1 ml) of the sample. A significant decrease in concentration indicates instant precipitation. After 3 days of agitation **(4)**, the measured concentration exhibited greater standard deviation values due to light scattering from the precipitated particles. However, the centrifuged sample showed a more consistent concentration range. By converting the pH of the aggregated sample back to neutral **(5)**, the concentration of the initial sample was regained.

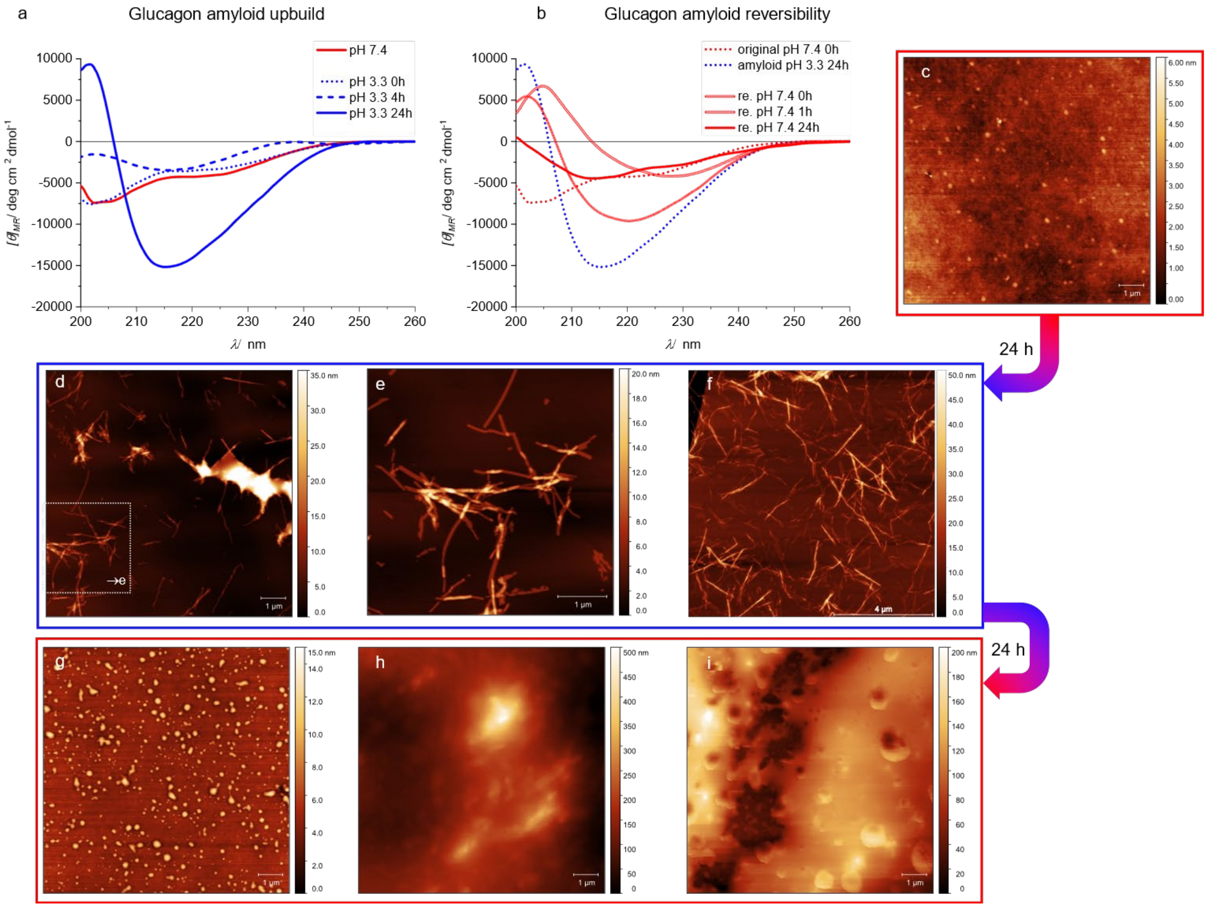

Supplementary Figure 18: Amyloid formation and reversibility of full-length glucagon. **a)** Amyloid formation of 1 mg ml^{-1} glucagon samples at *pH* 3.3 in the presence of 100 mM NaCl was monitored by CD spectroscopy. The sample was incubated at 37°C and stirred continuously with a magnetic stirrer. After one day, the amyloid formation was complete (blue line), as confirmed by the intense characteristic B-type spectra. **b)** To study the reversibility of fibril formation, we then changed the pH of the mature glucagon amyloid sample (blue dotted line) to 7.4 (triple red line). Immediately, the intensity of the B-type spectra began to decrease, and after one hour of incubation (double red line), the local maxima and minima of the spectrum shifted to higher wavelengths, specifically 201.5 nm to 205 nm and 215 nm to 228 nm, respectively. Even after 24 hours of incubation (red line), the spectrum did not completely return to its initial form (red dotted line), but the initial and reversed spectra resembled each other. We confirmed the reversibility of glucagon amyloid fibrils by AFM measurements. **c)** Initially, we observed a sedimented molecular layer (c), but after 24 hours of agitation, uniform glucagon fibrils appeared with an average height of 8-12 nm (**d-f**). Finally, the reversibility experiment followed by AFM confirmed that the fibrils disappeared completely after 24 hours of incubation at *pH* 7.4 (**g-i**).

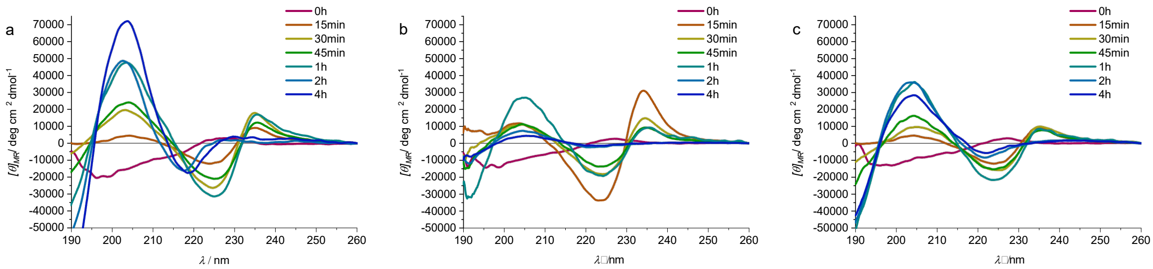

Supplementary Figure 19: Amyloid aggregation of the APR^{Tc5b} - LYIQWL oligopeptide (*c*= 0.19 mM) at *pH* 5.65 of increased ionic (NaCl) strengths. **a)** Control sample, **b)** 5 mM NaCl, **c)** 15 mM NaCl. The increased noise ratio of the detected CD-curves below 190 nm is explained by the elevated ionic strength of the sample.

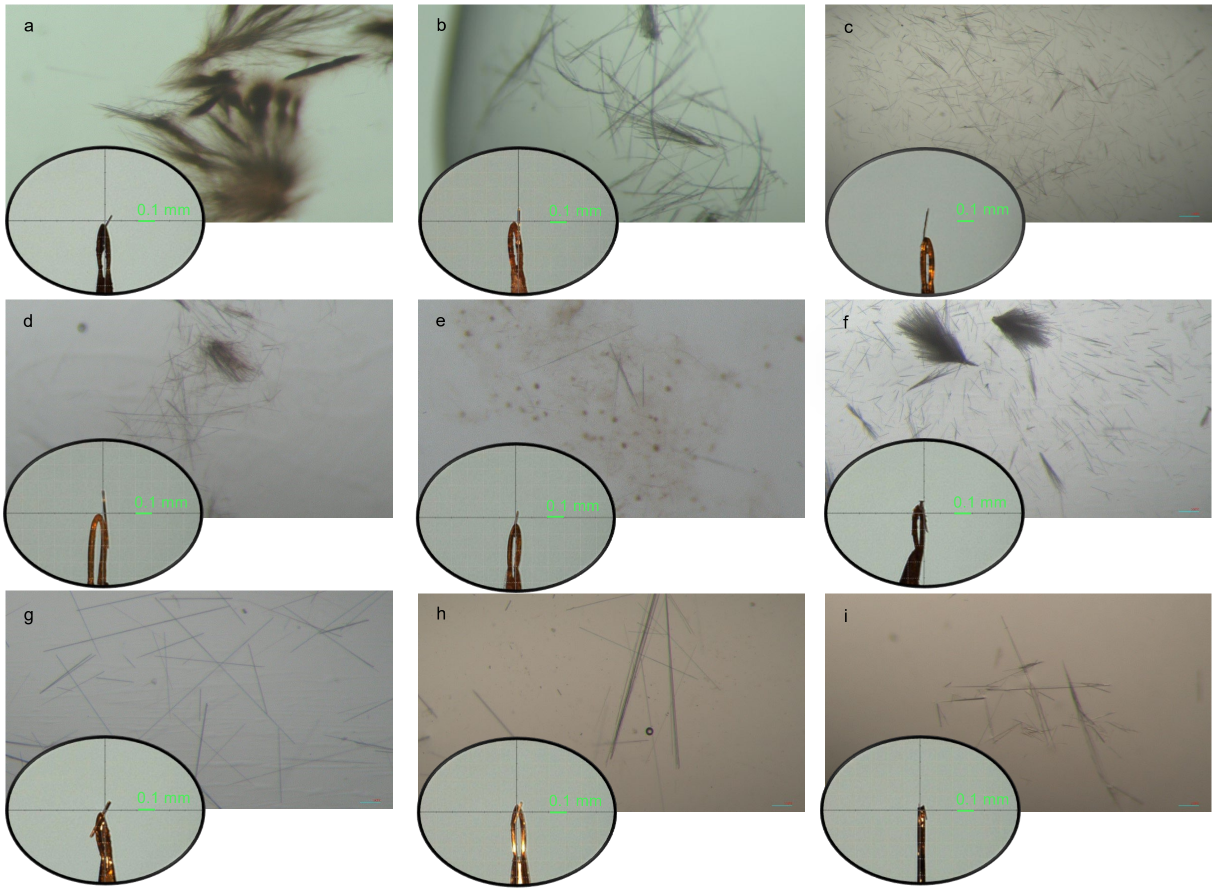

Supplementary Figure 20: Representative microscopic images of the amyloid-like microcrystals in the crystallization vial (background) and mounted on loops (front ovals) for the crystallographic measurements. 8-10 µl aliquots were pipetted from the vials containing 200 µl crystallization solution and microcrystals. This was repeated 2-4 times during crystal harvesting with all aliquots from the same experiment containing crystals of similar size and quality. **a)** DFINWL, **b)** pEFIAWL, **c)** Ac-EFIAWL, **d)** LFIEWL polymorph A, **e)** LFIEWL polymorph B, **f)** LYIQWL polymorph A, **g)** LYIQWL polymorph B, **h)** LYIQWL polymorph C and **i)** LYIQWL polymorph D.

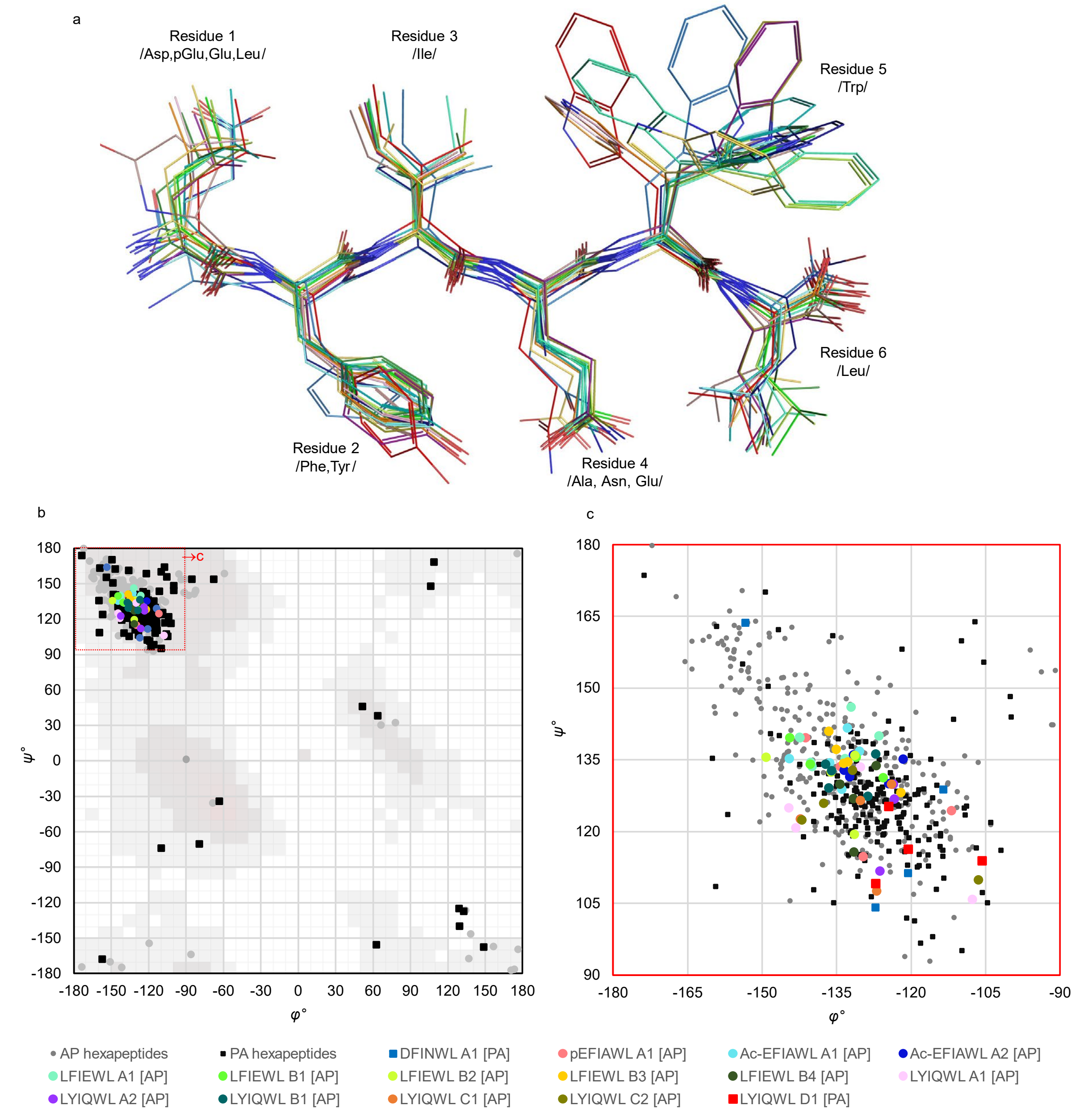

Supplementary Figure 21: Dihedral analysis of APR hexapeptides. **a)** Aligned single β-stranded backbone conformers found in amyloid-like crystal polymorphs of this study. **b-c)** The Ramachandran diagram was constructed by plotting the backbone torsion angles of each hexapeptide amyloid-like crystal structure available in the PDB in shades of gray, with the APR hexapeptides of the glucagon family represented in color. The exact dihedral values can be found in **Supplementary Table 2**, while the list of all structures downloaded for the construction of the diagram is in Supplementary Table 3. Parallel (PA) β-strands are represented by squares, while antiparallel (AP) strands are represented by circles. Our conclusion is that there is no sharp boundary between the distribution of PA and AP backbone-related in space spanned by ϕ and ψ angles. The distribution of PA torsion angles covers a well-defined and smaller area with an average of ϕ^{PA}= -126.6° / ψ^{PA}= +127.1°, while those of AP are ϕ^{AP}= -133.8° / ψ^{AP}= +135.9°. However, it should be noted that AP torsion angles are more dispersed and greatly overlap with those arranged in a parallel fashion. The AP-arranged APRs of the glucagon family occur at the boundary of the AP and PA sets, while the distribution of PAs is more distinguishable from the rest of the AP structures due to their torsion angles, which are typical of parallel structures.

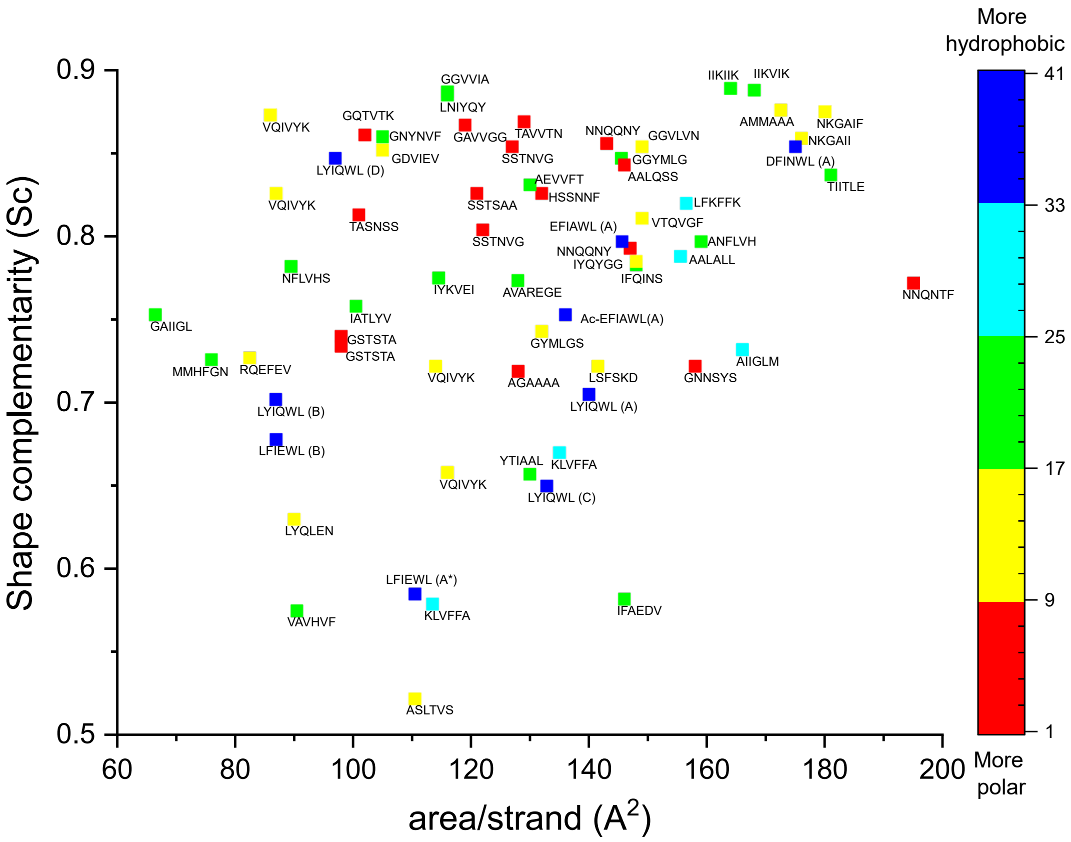

Supplementary Figure 22: Area of pairing dry interfaces plotted as a function of the shape complementarity (Sc) for 64 amyloid-like hexapeptides (**Supplementary Table 3**). The values of Sc and area (Å^2) were calculated by an in-house developed Pymol script. The areas are normalized for a single β-strand. The color mapping is based on the hydrophobic character of the hexapeptides defined by Thermo Fisher peptide online analyzing tool (https://www.thermofisher.com/hu/en/home/life-science/protein-biology/peptides-proteins/custom-peptide-synthesis-services/peptide-analyzing-tool.html). * Note that the structural ambiguity of LFIEWL polymorph A doesn't allow us to estimate precisely the Sc and contacting area values, due to multiple backbone and side chain conformers.

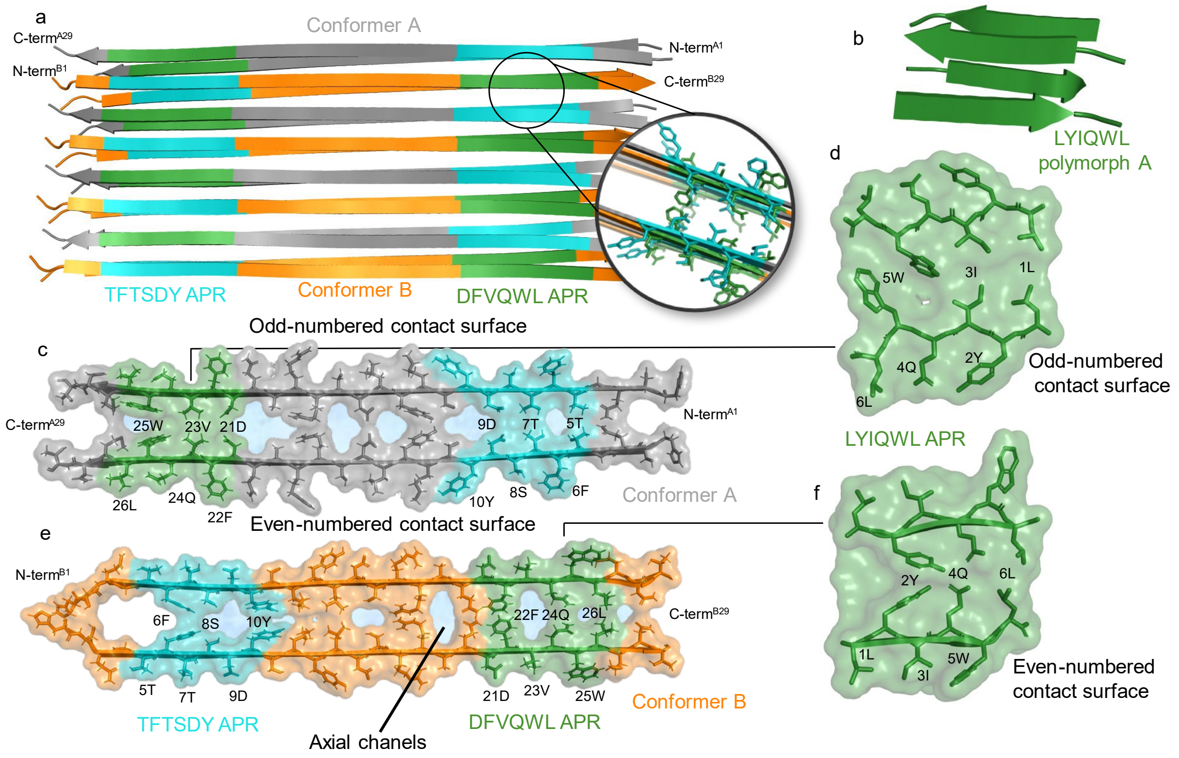

Supplementary Figure 23: The topology of the APR hexapeptides was compared with that of the full-length antiparallel glucagon amyloid fibril. Despite the high degree of sequence homology, it was not possible to grow sufficiently large crystals of the APR^{gluc} and APR^{GIP} hexapeptides. However, the information gathered on their amyloid formation suggests that they have similar spectral and morphological properties to the full-length glucagon amyloid fibril. **a)** The APR sequences of DFVQWL (green) and TSTSDY (turquoise) in the glucagon fibril (PDB ID: 6NZN) form a mixed antiparallel β-sheet in which the **c)** odd-numbered side chains of conformer A and **e)** the even-numbered side chains of conformer B form an alternating molecular zipper perpendicular to the fibril axis. This may provide a possible molecular explanation for why amyloid-like crystals could not be obtained from DFVQWL and DFVNWL separately, as they may prefer a similar mixed β-sheet structure. The APRs of exendin-4, GLP-1, and 2 were able to crystallize alone, suggesting that the full-length hormones may be packed differently into fibrils compared to antiparallel glucagon. **b)** The polymorphs A (and C) of APR^{Tc5b} have a contact surface pattern that follows an odd(**d**)/even(**e**) numbering scheme, similar to that of DFVQWL in antiparallel glucagon. Axial channels (**e-f**), shown in light blue along the fibril axis, allow solvents to reach gatekeeper residues that would otherwise be buried in the steric zipper. This accessibility allows side chain deprotonation, which facilitates fibril degradation.

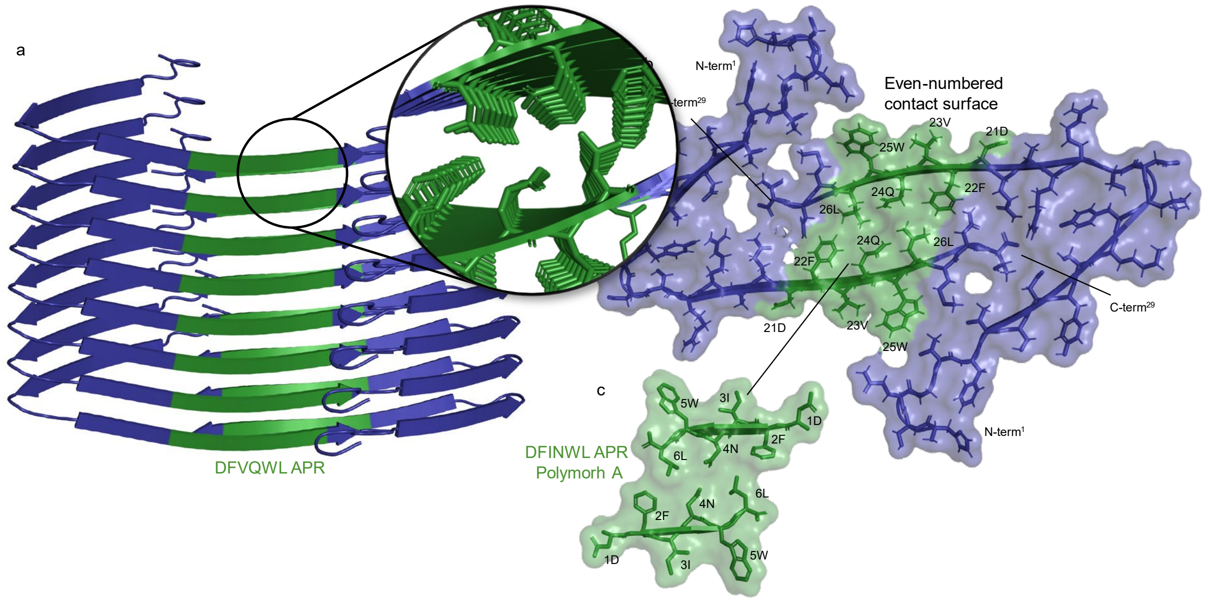

Supplementary Figure 24: The topology of the hexapeptide APRs was compared with that of the full-length parallel glucagon amyloid fibril. **a-b)** The APR sequence of DFVQWL (green) forms a central steric zipper of the class 1 topology in the parallel assembled glucagon fibril. (PDB ID: 7XM8) **c)** Interface 2 in polymorph A of APR^{GLP2} (DFINWL), which sequence is a closely related sequence to DFVQWL, displays similar sidechain interactions in the shifted contact surfaces between the even-numbered residues F-L, Q-Q, or N-N. The only noticeable difference is the length of the amide-side chains, which is also insignificant due to their comparable chemical properties and hydrogen bonding capacity.

a

APR sequences	t_R (min)	$m/z_{calculated}$ $[M+H]^+$	$m/z_{measured}$ $[M+H]^+$
EFIAWL	18.947	778.41	778.42
DFINWL	18.880	807.40	807.41
DFVNWL	18.553	793.39	793.40
DFVQWL	18.453	807.40	807.40
LFIEWL	19.720	820.46	820.48
LYIQWL	18.733	835.47	835.49

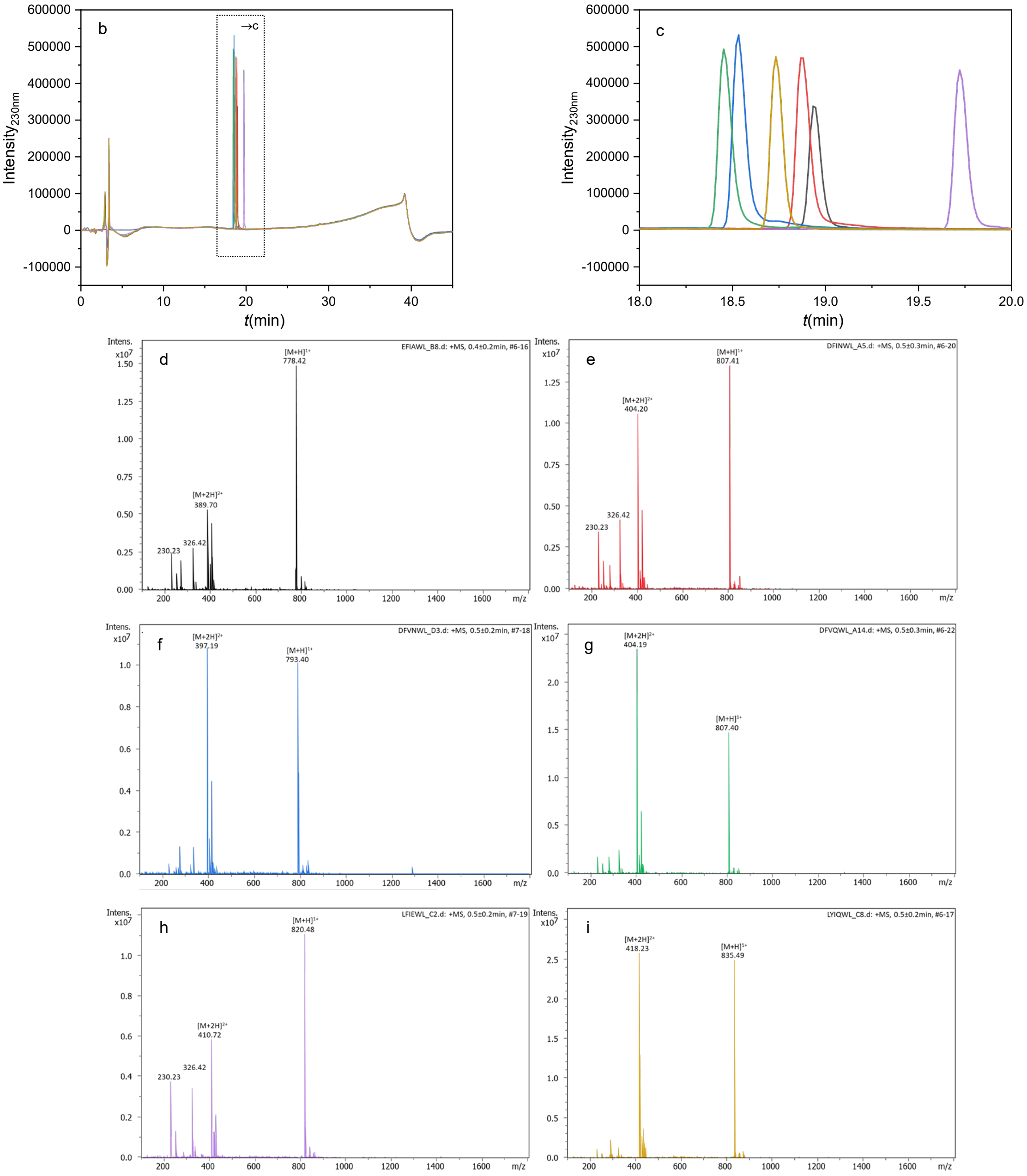

Supplementary Figure 25: Analytical characterization of the synthesized APR hexapeptides. **a)** Analytical HPLC retention times and the calculated and measured mass-to-charge ratios of the APR-s. **b-c)** Analytical HPLC chromatograms and the **d-i)** measured mass spectra of the unprotected hexapeptides. It should be noted that the m/z values of 230.23 and 326.42 are a result of general contamination of the local mass spectrometer and do not relate to the hexapeptides being studied.

Supplementary Table 1: X-ray data collection and refinement statistics. Data for the highest resolution shell are given in parentheses.

	DFINWL	pEFIAWL	Ac-EFIAWL
Data collection			
Unit cell: a,b,c (Å)	21.780,4.869,21.936	20.710,9.545,22.396	9.536, 42.631, 11.197
α,β,γ (°)	90,101.73,90	90, 92.74, 90	90, 95.68, 90
Space group	$P2_1$	C2	$P2_1$
Resolution range (Å)	16.96-1.55 (1.61-1.55)	11.19-1.30 (1.35-1.30)	10.78 – 1.50 (1.55 – 1.50)
No. of unique refl. / observed refl.	773 / 2226	1157 / 5529	1419 / 4259
<I / σI>	12.1 (2.2)	13.6 (3.9)	6.2 (1.2)
R_{meas}	0,067 (0.356)	0.106 (0.331)	0.135 (0.632)
Completeness (%)	98.35 (87.50)	99.83 (99.13)	97.46 (93.29)
CC(1/2)	0.999 (0.921)	0.995 (0.893)	0.995 (0.699)
Refinement			
Resolution range (Å)	16.96-1.55	11.19-1.30	10.78 – 1.50
R / R_{free} (No. of obs.)	0.168 (694) / 0.174 (74)	0.101 (1032) / 0.130 (121)	0.1511 (1270) / 0.1737 (140)
B factor of peptide / solvent (Å^2)	13.13 / 25.66	5.98 / 9.03	118 / 2
RMS dev. bond length (Å)	0,011	0,015	8.78 / 8.90
RMS dev. bond angles (°)	0,956	1,911	0.009
Ramachandran fav/all/disall	4 / 0 / 0	3 / 0 / 0	0.941
No. of non hydrogen atoms: peptide / solvent	58 / 1	55 / 2	8 / 0 / 0
PDB code	8ANJ	8ANK	8ONQ

	LFIEWL (polymorph A)	LFIEWL (polymorph B)	LYIQWL (polymorph A)
Data collection			
Unit cell: a,b,c (Å)	9.547, 23.404, 44.050	9.520, 14.292, 34.287	11.744,23.164,18.715
α,β,γ (°)	90, 90, 90	90.59, 93.30, 95.97	90,90.23,90
Space group	I222	P1	$P2_1$
Resolution range (Å)	20.67-1.24 (1.29-1.24)	14.21-1.75 (1.81-1.75)	14.56-1.25 (1.29-1.25)
No. of unique refl. / observed refl.	1514 / 12512	1803 / 5668	2829 / 18673
<I / σI>	9.5 (1.4)	6.4 (1.6)	9.2 (3.1)
R_{meas}	0.115 (0.904)	0.144 (0.799)	0.139 (0.468)
Completeness (%)	96.49 (63.51)	99.28 (99.48)	99.33 (97.26)
CC(1/2)	1.000 (0.977)	0.984 (0.700)	0.998 (0.777)
Refinement			
Resolution range (Å)	20.67-1.24	14.21-1.75	14.56-1.25
R / R_{free} (No. of obs.)	0.177 (1333) / 0.186 (149)	0.176 (1602) / 0.213 (178)	0.098 (2542) / 0.137 (275)
B factor of peptide / solvent (Å^2)	15.13 / 25.13	14.52 / 17.83	5.78 / 6.97
RMS dev. bond length (Å)	0,014	0,018	0,012
RMS dev. bond angles (°)	1,544	1,779	1,084
Ramachandran fav/all/disall	4 / 0 / 0	16 / 0 / 0	8 / 0 / 0
No. of non hydrogen atoms: peptide / solvent	81 / 6	241 / 3	120 / 23
PDB code	8ANN	8ANL	8ANH

	LYIQWL (polymorph B)	LYIQWL (polymorph C)	LYIQWL (polymorph D)
Data collection			
Unit cell: a,b,c (Å)	9.492,21.675,23.686	11.719,23.467,18.762	4.875,23.713,40.764
α,β,γ (°)	90,90,90	90, 90,04, 90	90,90,90
Space group	$P2_12_12_1$	$P2_1$	$P2_12_12_1$
Resolution range (Å)	15.99-0.90 (0.93-0.90)	18.76-1.35 (1.40-1.35)	20.50-1.50
No. of unique refl. / observed refl.	3952 / 22002	2253 / 11098	895 / 3631
<I / σI>	9.1 (2.3)	7.0 (2.2)	13.5 (3.8)
R_{meas}	0.128 (0.672)	0.168 (0.745)	0.066 (0.204)
Completeness (%)	99.92 (99.75)	98.13 (94.69)	95.01 (65.59)
CC(1/2)	0.999 (0.761)	0.998 (0.756)	0.998 (0.978)
Refinement			
Resolution range (Å)	15.99-0.90	18.76-1.35	20.50-1.50
R / R_{free} (No. of obs.)	0.130 (3541) / 0.145 (393)	0.114 (2023) / 0.152 (221)	0.084 (812) / 0.095 (81)
B factor of peptide / solvent (Å^2)	4.97 / 8.25	6.59 / 10.03	4.01 / 11.02
RMS dev. bond length (Å)	0,009	0,013	0,007
RMS dev. bond angles (°)	1,155	1,410	1,236
Ramachandran fav/all/disall	4 / 0 / 0	8 / 0 / 0	4 / 0 / 0
No. of non hydrogen atoms: peptide / solvent	69 / 5	120 / 26	64 / 3
PDB code	8ANM	8ANI	8ANG

Supplementary Table 2: The φ and ψ backbone torsion angles of the APR hexapeptides are presented **in Supplementary Figure 21.** It is important to note that some of the selected residues adopt more than one conformational state, which is a sign of backbone plasticity.

Conformer			Residue 1 Φ° / Ψ°	Residue 2 Φ° / Ψ°	Residue 3 Φ° / Ψ°	Residue 4 Φ° / Ψ°	Residue 5 Φ° / Ψ°	Residue 6 Φ° / Ψ°
Polymorph								
DFINWL	A	1	- / 150.4°	-153.4° / 163.7°	-113.5° / 128.8°	-127.2° / 104.1°	-120.6° / 111.3 °	-102.6° / -
pEFIAWL	A	1	- / 156.7°	-111.8 ° / 124.3°	-134.2° / 133.6°	-141.2° / 139.6°	-129.6° / 114.7 °	-83.8° / -
Ac-EFIAWL	A	1	-144.5° / 135.2°	-136.3° / 134.3°	-130.4° / 136.8°	-132.8° / 141.6°	-133.9° / 128.8°	-144.5° / -
	A	2	-124.5° / 130.0°	-121.6° / 135.1°	-132.3° / 131.4°	-131.6° / 136.0°	-133.4° / 132.8°	-124.5° / -
LFIEWL	A	1	- / 128.1°	-133.3° / 135.2°	-140.2° / 134.4°<br>-139.2° / 133.6°	-142.4° / 139.7°<br>-142.3° / 139.6°	-126.4° / 140.0°<br>-132.0° / 146.0°	-148.6° / -
	B	1	- /130.0°	-125.6° / 131.2°	-131.0° / 135.5°	-140.3° / 134.0°	-144.4° / 139.6°	-151.3° / -
		2	- / 135.3°	-149.2° / 135.5°	-136.1° / 132.4°	-131.3° / 135.9°	-131.4° / 119.4 °	-125.0° / -
		3	- / 128.1°	-122.0° / 128.1°	-132.7° / 134.5°<br>-135.0° / 137.2°	-136.5° / 140.9°	-133,5° / 134.2°	-148.6° / -
		4	- / 133.5°	-134.3° / 129.9°	-131.6° / 126.9°	-127.0° / 133.8°	-131.5° / 115.7 °	-119.6° / -
LYIQWL	A	1	- / 149.9°	-123.3° / 126.8°	-123.5° / 129.9°	-142.2° / 122.6°	-126.2° / 111.7 °	-76.9° / -
		2	- / 137.0°	-143.2° / 120.7°	-130.2° / 133.5°	-144.6° / 124.9°	-107.6° / 105.8°	-97.9° / -
	B	1	- / 135.2°	-136.5° /129.1°	-127.1° / 136.2°	-137.2° / 134.0°<br>-136.0° / 132.7°	-128. ° 7 / 127.3°	-130.7° / -
	C	1	- / 155.8°	-130.2° /126.5°	-123.9° / 129.9°	-142.2° / 122.6°	-126.9° / 107.5°	-72.8° / -
		2	- / 132.1°	-137.6° / 125.9°	-131.8° / 132.8°	-142.0° / 122.4°	-106.4° / 109.9°	-101.4° / -
	D	1	- / 130.4°	-120.5° / 116.3 °	-105.7° / 113.8 °	-127.1° / 109.1°	-124.5° / 125.2°	-130.7° / -

Supplementary Table 3: The list of amyloidogenic hexapeptides with their respective PDB references used in this work. The exact values of SC, area/strand, and hydrophobicity are given for the hexapeptides shown in **Supplementary Figure 22.** (* Note that the structural ambiguity of LFIEWL polymorph A did not allow us to accurately estimate the Sc and contact area values, due to multiple backbone and side chain conformers. In order to approximate them, a two chain model was created with one chain containing only the A and the other the B alternative conformers.)

PDB code	sequence	SC	area/strand (Å^2)	hydrophobicity	PDB code	sequence	SC	area/strand (Å^2)	hydrophobicity
1YJO	NNQQNY	0.856	143.0	2.9	5WHN	NFGAFS	-	-	-
2OKZ	MVGGVV	-	-	-	5WHP	NFGTFS	-	-	-
2OL9	SNQNNF	-	-	-	5WIA	GNNSYS	0.722	158.0	2.7
2OMP	LYQLEN	0.630	90.0	16.3	5WKB	NFGEFS	-	-	-
2OMQ	VEALYL	-	-	-	5WMJ	KVWGSI	-	-	-
2ON9	VQIVYK	0.722	114.0	15.7	6BWZ	SYSGYS	-	-	-
2ONA	MVGGVV	-	-	-	6BXX	GYNGFG	-	-	-
2ONV	GGVVIA	0.887	116.0	17.0	6BZP	STGGYG	-	-	-
2ONW	SSTSAA	0.826	121.0	1.9	6C3F	IYKVEI	0.775	114.5	21.5
2Y29	KLVFFA	-	-	-	6C3G	KALGIS	-	-	-
2Y2A	KLVFFA	0.579	113.5	31.2	6C3S	YTIAAL	0.657	130.0	20.3
2Y3J	AIIGLM	0.732	166.0	29.3	6C3T	AADTWE	-	-	-
3DG1	SSTNVG	0.854	127.0	3.0	6C88	VAVHVF	0.575	90.5	20.5
3FOD	AILSST	-	-	-	6CB9	AALQSS	0.843	146.0	4.8
3FPO	HSSNNF	0.826	132.0	7.3	6CEW	AMMAAA	0.876	172.5	10.4
3FR1	NFLVHS	0.782	89.5	20.3	6DIX	NFVFGT	-	-	-
3FTR	SSTNVG	0.804	122.0	3.3	6DJ0	ASLTVS	0.522	110.5	11.1
3FVA	NNQNTF	0.772	195.0	7.7	6EEX	GSTSTA	0.734	98.0	2.2
3LOZ	LSFSKD	0.722	141.5	12.9	6FG4	IIKVIK	0.888	168.0	20.5
3NHC	GYMLGS	0.743	132.0	16.6	6FGR	IIKIIK	0.889	164.0	22.7
3NHD	GYVLGS	-	-	-	6FHD	LFKFFK	0.820	156.5	31.2
3NVE	MMHFGN	0.726	76.0	17.6	6G8C	IYQYGG	0.785	148.0	10.8
3NVF	IIHFGS	-	-	-	6G8D	LNIYQY	0.885	116.0	20.2
3NVG	MIHFGN	-	-	-	6G8E	VTQVGF	0.811	149.0	15.8
3OVJ	KLVFFA	0.670	135.0	30.2	6G9G	TASNSS	0.813	101.0	0.8
3OVL	VQIVYK	0.658	116.0	15.7	6M9I	GSTSTA	0.740	98.0	2.2
3OW9	KLVFFA	-	-	-	6N4P	RQEFEV	0.727	82.5	15.1
3PPD	GGVLVN	0.854	149.0	15.2	6ODG	SVQIVY	-	-	-
3PZZ	GAIIGL	0.753	66.5	22.7	6PQ5	AGAAAA	0.719	128.0	2.2
3Q2X	NKGAII	0.859	176.0	13.3	6PQA	GAVVGG	0.867	119.0	6.8
3SGS	GDVIEV	0.852	105.0	16.6	6RHA	NTVTFN	-	-	-
4NP8	VQIVYK	0.873	86.0	15.7	6RHB	IATLYV	0.758	100.5	23.0
4R0P	IFQINS	0.783	148.0	19.2	6RHD	TSYVGV	-	-	-
4RP7	TIITLE	0.837	181.0	23.5	6UOP	AVAAGA	-	-	-
4TUT	GGYMLG	0.847	145.5	17.7	6WPQ	GNYNVF	0.860	105.0	17.5
4UBY	GGYVLG	-	-	-	7LTU	AALALL	0.788	155.5	25.7
4UBZ	GGYLLG	-	-	-	7LUX	AALALL	-	-	-
4XFN	AEVVFT	0.831	130.0	19.7	7LUZ	GQTVTK	0.861	102.0	4.0
4XFO	TAVVTN	0.869	129.0	8.1	7LV2	GSQASS	-	-	-
5E5X	ANFLVH	0.797	159.0	21.4					
5E5Z	LVHSSN	-	-	-	8ANJ	DFINWL (A)	0.854	175.0	36.4
5K2E	NNQQNY	-	-	-	8ANK	EFIAWL (A)	0.797	145.6	34.5
5K2F	NNQQNY	0.793	147.0	2.9	8ONQ	Ac-EFIAWL (A)	0.753	136.0	33.4
5K7N	VQIVYK	0.826	87.0	15.7	8ANN	LFIEWL (A*)	0.585	110.5	40.1
5TXD	NKGAIF	0.875	180.0	16.2	8ANL	LFIEWL (B)	0.678	87.0	40.1
5TXH	IFAEDV	-	-	-	8ANH	LYIQWL (A)	0.705	140.0	36.5
5TXJ	IFAEDV	0.582	146.0	19.8	8ANM	LYIQWL (B)	0.702	86.9	36.5
5V5C	VQIINK	-	-	-	8ANI	LYIQWL (C)	0.650	132.9	36.5
5W50	LIIKGI	-	-	-	8ANG	LYIQWL (D)	0.847	97.0	36.5

Supplementary Table 4: Structure-based computational pK_a values were determined using PROPKA 3.5[3,4] The input structures used for the calculations were as follows: **a)** The asymmetric unit of the crystal structures. **b)** The modified asymmetric unit of the pEFIAWL crystal (denoted by *), where the pGlu residue was mutated to Glu with the most common rotamer of the side chain. **c)** Identical hexapeptide APR sequences were extracted from the structural assemblies of full-length glucagon fibrils (DFVQWL). In addition, an *in-silico* mutation of Q4N was introduced to generate the DFVNWL monomeric unit.

APR	conformer/model	pK_a C-term.	pK_a side chain	pK_a N-term.
LYIQWL	A1	3.41	10.46	8.46
	A2	2.74	10.28	7.43
	B1	3.37	10.25	7.93
	C1	3.14	10.48	8.49
	C2	2.13	10.31	7.89
	D1	3.27	9.59	7.92
	average	3.01	10.23	8.02
LFIEWL	A1	3.37	4.65	7.92
	B1	3.32	4.71	8.52
	B2	2.66	4.72	8.09
	B3	3.26	4.7	8.78
	B4	2.31	4.15	7.7
	average	2.98	4.59	8.2
Ac-EFIAWL	A1	2.76	4.56	n.d.
	A2	2.74	4.64	n.d.
	average	2.75	4.6	n.d.
(p)EFIAWL	pEFIAWL 1	3.38	n.d.	n.d.
	EFIAWL model*	3.38	4.55	7.88
DFINWL	A1	3.34	3.84	7.91
DFVNWL	6nzn2B_N4-mcmm	3.48	3.88	7.84
	6nzn3K_N4-mcmm	3.24	3.97	8
	7xm8_allSch-mcmm	3.25	3.83	7.79
	average	3.32	3.89	7.88
DFVQWL	6nzn_ver2B	3.5	3.87	7.84
	6nzn_ver3K	3.25	3.97	7.89
	7xm8_L	3.48	3.88	7.84
	average	3.41	3.91	7.86

Supplementary Discussion

This additional discussion focuses on the applicability of approximate acidic dissociation constants (pK_a) used in our work. The exact pK_a values of the of individual amino acids depend heavily on the local chemical environment of the specific functional group, which is influenced by the tertiary structure and various chemical interactions within the molecule. It is highly recommended to experimentally determine the individual pK_a values to accurately assess the distribution of differently charged species as a function of pH, this however, is quite a challenge in a number of cases. The most accessible approaches, such as Z-potential titration measurements for example, provide information about the overall charge of the system, yielding macroscopic pK_a values, but they are not suitable for determining microscopic pK_a values that accurately describe the protonation state of all functional groups of a polypeptide. NMR spectroscopy can also be utilized, as it can accurately determine individual pK_a values by observing changes in chemical shifts as a function of pH, if complete assignment of the studied systems can be carried out. Unfortunately, this could not be achieved in case of all six hexapeptides studied here due to their tendency to aggregate, which hinders accurate measurements especially in the acidic pH range. A reasonable initial approximation in case of short, unstructured segments – such as the monomeric form of APR hexapeptides – would be the simple application of “general” microscopic pK_a values which can be readily found in the literature. However, it should be noted that even in case of these moderately sized segments, significant differences may arise in the pK_a of acidic gatekeeper residues depending on their position; for example, close to the N-terminus these residues can interact intramolecularly with the N-terminal amine, thereby altering the pK_a values of both functional groups.

To provide a more reliable values that is able to account for these differences, we determined the pK_a values using the PROPKA approach, as described in the Material and Methods section.

Supplementary References

[1] Handbook of Hormones - 2nd Edition. https://www.elsevier.com/books/handbook-of-hormones/ando/978-0-12-820649-2. (ISBN: 978-0-12-820649-2)

[2] Linding R, Schymkowitz J, Rousseau F, Diella F, Serrano LA comparative study of the relationship between protein structure and beta-aggregation in globular and intrinsically disordered proteins *J Mol Biol* 345-353, (2004)

[3] Olsson, M. H. M., Søndergaard, C. R., Rostkowski, M. & Jensen, J. H. PROPKA3: Consistent Treatment of Internal and Surface Residues in Empirical pKa Predictions. *J. Chem. Theory Comput.* **7**, 525–537 (2011).

[4] Søndergaard, C. R., Olsson, M. H. M., Rostkowski, M. & Jensen, J. H. Improved Treatment of Ligands and Coupling Effects in Empirical Calculation and Rationalization of pKa Values. *J. Chem. Theory Comput.* **7**, 2284–2295 (2011).
