# Supplementary material for: Polymorphic amyloid nanostructures of hormone peptides involved in glucose homeostasis display reversible amyloid formation
Source: Nat Commun. 2023 Aug 1;14:4621. doi: 10.1038/s41467-023-40294-x (PMC10394066; doi:10.1038/s41467-023-40294-x)
Supplement: Supplementary file 3 — Reporting Summary [file 41467_2023_40294_MOESM3_ESM.pdf]

nature portfolio

Corresponding author(s): Prof. András Perczel

Last updated by author(s): Jul 4, 2023

Reporting Summary

Nature Portfolio wishes to improve the reproducibility of the work that we publish. This form provides structure for consistency and transparency in reporting. For further information on Nature Portfolio policies, see our Editorial Policies and the Editorial Policy Checklist.

Statistics

For all statistical analyses, confirm that the following items are present in the figure legend, table legend, main text, or Methods section.

n/a	Confirmed	
☐	☒	The exact sample size (*n*) for each experimental group/condition, given as a discrete number and unit of measurement
☐	☒	A statement on whether measurements were taken from distinct samples or whether the same sample was measured repeatedly
☒	☐	The statistical test(s) used AND whether they are one- or two-sided<br>*Only common tests should be described solely by name; describe more complex techniques in the Methods section.*
☒	☐	A description of all covariates tested
☒	☐	A description of any assumptions or corrections, such as tests of normality and adjustment for multiple comparisons
☐	☒	A full description of the statistical parameters including central tendency (e.g. means) or other basic estimates (e.g. regression coefficient) AND variation (e.g. standard deviation) or associated estimates of uncertainty (e.g. confidence intervals)
☒	☐	For null hypothesis testing, the test statistic (e.g. *F*, *t*, *r*) with confidence intervals, effect sizes, degrees of freedom and *P* value noted<br>*Give P values as exact values whenever suitable.*
☒	☐	For Bayesian analysis, information on the choice of priors and Markov chain Monte Carlo settings
☒	☐	For hierarchical and complex designs, identification of the appropriate level for tests and full reporting of outcomes
☒	☐	Estimates of effect sizes (e.g. Cohen's *d*, Pearson's *r*), indicating how they were calculated

Our web collection on statistics for biologists contains articles on many of the points above.

Software and code

Policy information about availability of computer code

Data collection	OPUS 6.5, Jasco Spectra Manager v.2.0, Nanosurf control software C3000 3.10.4, CrysAlisPro v 1.171.42.58a
Data analysis	Origin2020b, Excel 2016-2020, Gwyddion 2.62, Jasco Spectra Manager v.2.0, Phenix 1.20.1, Coot 0.9.8.5, CCP4 8.0, PyMOL 2.5.5, Thermo Fisher peptide analyzing tool, PROPKA 3.5, Schrödinger Release 2023-2: MacroModel, Schrödinger

For manuscripts utilizing custom algorithms or software that are central to the research but not yet described in published literature, software must be made available to editors and reviewers. We strongly encourage code deposition in a community repository (e.g. GitHub). See the Nature Portfolio guidelines for submitting code & software for further information.

Data

Policy information about availability of data

All manuscripts must include a data availability statement. This statement should provide the following information, where applicable:

- Accession codes, unique identifiers, or web links for publicly available datasets
- A description of any restrictions on data availability
- For clinical datasets or third party data, please ensure that the statement adheres to our policy

Structural coordinates have been deposited online in the Protein Data Bank under accession code PDB 8ANJ, 8ANK, 8ONQ, 8ANN, 8ANL, 8ANH, 8ANM, 8ANI, 8ANG. Structures under accession code 6NZN and 7XM8 were used in the discussion of this study.

March 2021

Human research participants

Policy information about studies involving human research participants and Sex and Gender in Research.

Reporting on sex and gender	N/A
Population characteristics	N/A
Recruitment	N/A
Ethics oversight	N/A

Note that full information on the approval of the study protocol must also be provided in the manuscript.

Field-specific reporting

Please select the one below that is the best fit for your research. If you are not sure, read the appropriate sections before making your selection.

☒ Life sciences ☐ Behavioural & social sciences ☐ Ecological, evolutionary & environmental sciences

For a reference copy of the document with all sections, see nature.com/documents/nr-reporting-summary-flat.pdf

Life sciences study design

All studies must disclose on these points even when the disclosure is negative.

Sample size	No statistical calculations were necessary to determine the sample sizes for this study. Since it is a structural/biochemical study that does not involve animal or human experiments, the sample sizes were not predetermined based on statistical considerations. Concentrations were chosen as the followings: a) CD measurements: The concentration range was chosen so that no further dilution was needed prior to CD measurements, and the measured absorbance of the individual samples should have been smaller than ABS = 1.1 b) FTIR measurements: The concentrations were determined after a discussion with experts in the field of FTIR to ensure that appropriate intensity was achieved. C) ThT: We adjusted the concentration to fall within the concentration range of the CD measurements.
Data exclusions	ThT: Data points were not recorded after 600 minutes because amyloid formation had already completed. No changes in trend were observed in the intensity values of the blank and ThT negative neutral samples in the data that were discarded after 600 minutes. CD of glucagon reversibility: (Supplemetary Figure 18.) Spectra were truncated from 185 nm to 200 nm because the increased noise level emerged due to the increased ion strength.
Replication	All measurements were carried on once, except ThT, in this case, three parallels were recorded. Preliminary experiments were conducted to determine the appropriate experimental conditions, e.g. setting the sensitivity level of the PMT in the Multiplate Reader. The results of these preliminary experiments are not included this study.
Randomization	Images were taken from random locations on the surface during the AFM measurements. Randomization is not relevant in biochemical/structural studies.
Blinding	Blinding was not relevant in this study because the biochemical nature of the topic.

Reporting for specific materials, systems and methods

We require information from authors about some types of materials, experimental systems and methods used in many studies. Here, indicate whether each material, system or method listed is relevant to your study. If you are not sure if a list item applies to your research, read the appropriate section before selecting a response.

Materials & experimental systems

n/a	Involved in the study
☒	☐ Antibodies
☒	☐ Eukaryotic cell lines
☒	☐ Palaeontology and archaeology
☒	☐ Animals and other organisms
☒	☐ Clinical data
☒	☐ Dual use research of concern

Methods

n/a	Involved in the study
☒	☐ ChIP-seq
☒	☐ Flow cytometry
☒	☐ MRI-based neuroimaging
